# Genome-wide and high-density CRISPR-Cas9 screens identify point mutations in *PARP1* causing PARP inhibitor resistance

**DOI:** 10.1038/s41467-018-03917-2

**Published:** 2018-05-10

**Authors:** Stephen J. Pettitt, Dragomir B. Krastev, Inger Brandsma, Amy Dréan, Feifei Song, Radoslav Aleksandrov, Maria I. Harrell, Malini Menon, Rachel Brough, James Campbell, Jessica Frankum, Michael Ranes, Helen N. Pemberton, Rumana Rafiq, Kerry Fenwick, Amanda Swain, Sebastian Guettler, Jung-Min Lee, Elizabeth M. Swisher, Stoyno Stoynov, Kosuke Yusa, Alan Ashworth, Christopher J. Lord

**Affiliations:** 1 The CRUK Gene Function Laboratory, The Institute of Cancer Research, London, SW3 6JB UK; 2 Breast Cancer Now Toby Robins Research Centre, The Institute of Cancer Research, London, SW3 6JB UK; 3 Institute of Molecular Biology “Roumen Tsanev”, Bulgarian Academy of Sciences, Sofia, 1113 Bulgaria; 4 University of Washington School of Medicine, 1959 NE Pacific St, Seattle, WA 98195 USA; 5 Divison of Structural Biology, The Institute of Cancer Research, London, SW3 6JB UK; 6 Tumour Profiling Unit, The Institute of Cancer Research, London, SW3 6JB UK; 7 Center for Cancer Research, National Cancer Institute, Bethesda, MD 20892 USA; 8 Wellcome Trust Sanger Institute, Hinxton, Cambridgeshire CB10 1SA UK; 9 UCSF Helen Diller Family Comprehensive Cancer Center, 1450 3rd St, San Francisco, CA 94158 USA

## Abstract

Although PARP inhibitors (PARPi) target homologous recombination defective tumours, drug resistance frequently emerges, often via poorly understood mechanisms. Here, using genome-wide and high-density CRISPR-Cas9 “tag-mutate-enrich” mutagenesis screens, we identify close to full-length mutant forms of PARP1 that cause in vitro and in vivo PARPi resistance. Mutations both within and outside of the PARP1 DNA-binding zinc-finger domains cause PARPi resistance and alter PARP1 trapping, as does a PARP1 mutation found in a clinical case of PARPi resistance. This reinforces the importance of trapped PARP1 as a cytotoxic DNA lesion and suggests that PARP1 intramolecular interactions might influence PARPi-mediated cytotoxicity. *PARP1* mutations are also tolerated in cells with a pathogenic *BRCA1* mutation where they result in distinct sensitivities to chemotherapeutic drugs compared to other mechanisms of PARPi resistance (*BRCA1* reversion, *53BP1*, *REV7* (*MAD2L2*) mutation), suggesting that the underlying mechanism of PARPi resistance that emerges could influence the success of subsequent therapies.

## Abstract (web summary)

The mechanisms of PARP inhibitor (PARPi) resistance are poorly understood. Here the authors employ a CRISPR mutagenesis approach to identify PARP1 mutants causing PARPi resistance and find that PARP1 mutations are tolerated in BRCA1 mutated cells, suggesting alternative resistance mechanisms.

## Introduction

Drugs targeting the poly-(ADP-ribose) polymerase (PARP) enzymes PARP1 and PARP2 cause synthetic lethality in tumour cells with homologous recombination (HR) defects, including those with loss-of-function mutations in the *BRCA1* or *BRCA2* tumour suppressor genes^1–3^. PARP1 acts as a DNA damage sensor, rapidly binding single- and double-stranded DNA breaks as they occur and then coordinating their repair by synthesising poly-(ADP-ribose) (PAR) chains on target proteins (PARylation)^4^. The rationale for using PARP inhibitors (PARPi) to treat HR-deficient cancers is based on the exquisite sensitivity of *BRCA1*- or *BRCA2*-defective cells to small-molecule PARPi, as well as the ability of *Parp1* gene silencing to selectively inhibit *Brca1*- or *Brca2*-defective cells^1, 2^. Subsequent experiments have revealed that in addition to inhibiting the catalytic activity of PARP1, most clinical PARPi cause cytotoxicity by trapping PARP1 at sites of DNA damage^5–7^, consistent with earlier observations that PARP1 displays a greater affinity for DNA breaks in the presence of toolbox PARPi^8, 9^. The PARP1 trapping potency of different inhibitors correlates with their cytotoxic potency, with talazoparib (BMN673, Pfizer) showing the greatest effect^5, 6^. Complete ablation of PARP1 expression by transposon-mediated mutagenesis or gene silencing in *BRCA1/BRCA2* wild-type (WT) cells results in extreme resistance to several PARPi^1, 5–7^.

The ability of some PARPi to trap PARP1 might be partially explained by the observation that PARP1 DNA binding is independent of its catalytic activity, while dissociation of PARP1 from DNA requires PARylation^10^. Recent structural studies have proposed a model of PARP1 binding to single-stranded DNA damage that takes into account a series of molecular interactions between different PARP1 protein domains^11–13^. In its non-DNA-bound state, a regulatory PARP1 helical domain (HD) is proposed to prevent catalytic activity. Upon PARP1 DNA binding (via N-terminal zinc-finger (ZnF) DNA-binding domains), an unfolding of the PARP1 helical region accompanies catalytic activation and PARP1 synthesises PAR chains on itself and other acceptor proteins in the vicinity^11–13^. These PARylation events recruit other DNA repair enzymes, such as XRCC1^14^, and act as a second messenger signalling the presence of DNA damage. The synthesis of highly negatively charged PAR chains on PARP1 is thought to also cause dissociation of PARP1 from DNA, presumably through a steric mechanism^10^.

Here we used CRISPR-Cas9 mutagenesis to investigate the mechanisms of PARPi toxicity in greater detail. We apply a focused mutagenesis approach to generate a large number of *PARP1* mutant alleles that cause resistance, identifying an axis of intramolecular communication in PARP1 that mediates PARPi toxicity. We isolate *PARP1* mutants from tumour cells with *BRCA1* exon 11 mutations and demonstrate that residual BRCA1 function in these cells allows tolerance of PARP1 loss of function, despite the synthetic lethal relationship between these genes. A *PARP1* mutation observed in a tumour from a PARPi-resistant patient prevents PARP1 trapping, suggesting that *PARP1* mutations that impair trapping could contribute to clinical PARPi resistance. Finally, we find that *PARP1* mutations caused a distinct set of drug sensitivities when compared to other known forms of PARPi resistance (loss of *REV7* (*MAD2L2*) or *TP53BP1*, or *BRCA1* reversion mutants), suggesting that knowledge of the molecular mechanism of resistance in individual patients could inform decisions on further treatment.

## Results

### In-frame *Parp1* deletions cause PARPi resistance

**Fig. 1:**
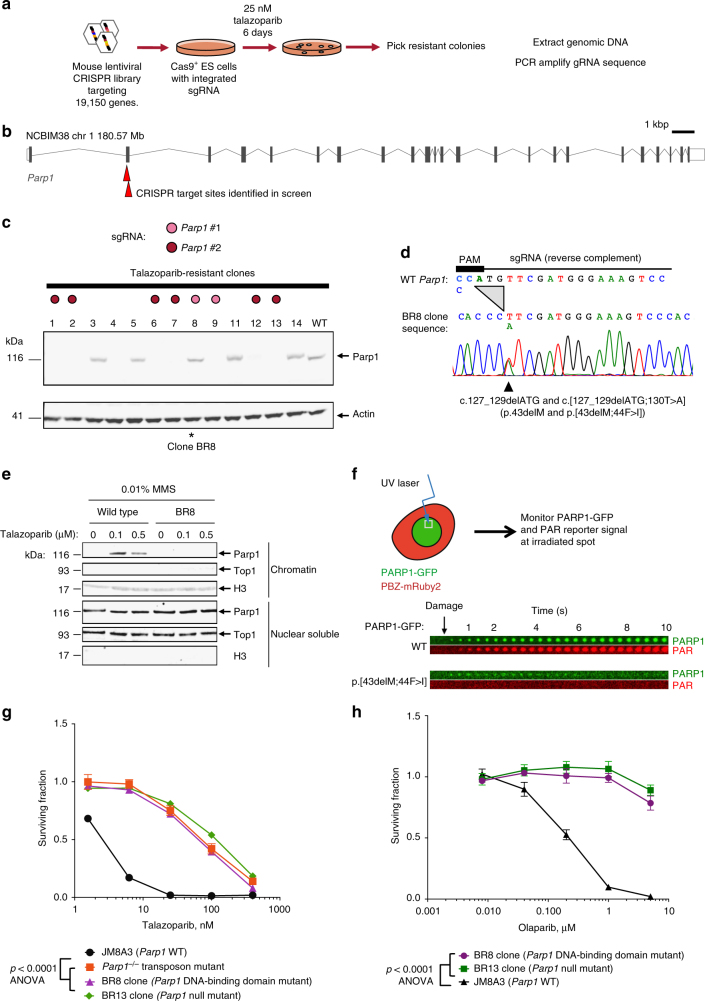
A genome-wide CRISPR screen for PARP inhibitor resistance identifies in-frame Parp1 mutants. **a** Experimental scheme. **b** Locations of *Parp1* guide RNA target sites in exon two of the mouse *Parp1* gene. **c** Parp1 western blot of lysates from talazoparib-resistant clones identified in the CRISPR screen. Individual clones are colour-coded according to sgRNA present (see key). Clones 1, 2, 6, 7, 9, 12 and 13 with *Parp1* sgRNAs have lost Parp1 protein expression, whilst *Parp1* sgRNA clone 8 (BR8, *) has retained Parp1 expression. **d** Clone BR8 has an in-frame *Parp1* deletion and a *Parp1* substitution mutation. Sanger sequencing trace of the *Parp1* sgRNA target site is shown, illustrating a 3 bp deletion on both alleles and a heterozygous c.130T>A substitution mutation (p.44F>I) close to the CRISPR PAM site. **e** Parp1 is not trapped in the chromatin fraction by PARP inhibitor in the BR8 clone. Western blots illustrating Parp1 in the chromatin and nuclear soluble fractions of wild-type ES cells and *Parp1* mutant BR8 cells exposed to talazoparib. Data shown are representative of two experiments. **f** PARP1 protein with a p.[43delM;44F>I] mutation has impaired recruitment to damaged DNA and does not initiate PAR synthesis at damaged DNA. Localisation of PARP1-GFP to damaged DNA was estimated by visualising GFP signal at the microirradiated spot, as was the generation of PAR at the damaged site by use of a PAR-binding PBZ-mRuby2 probe (see schematic). The time course of PARP1-GFP and PBZ-mRuby2 signals from CAL51 *PARP1*^*–/–*^ cells transfected with PARP1-GFP (top) and PARP1-p.[43delM;44F>I]-GFP (bottom) are shown. “Damage” denotes time at which microirradiation was carried out. **g**, **h** Dose response curves illustrating that clone BR8 is resistant to both talazoparib (**g**) and olaparib (**h**). BR13 is an ES cell mutant with no Parp1 protein expression (see panel **c**); a transposon-mutagenised, *Parp1* null mutant^7^ is also shown (red). Clone BR8 vs. BR13 and *Parp1*^–/–^ transposon, *p* = ns, ANOVA. Parp1 mutant clones vs. wild-type cells, *p* < 0.0001, ANOVA. Mean of five replicates plotted, error bars show SD. Surviving fractions were calculated relative to DMSO-exposed cells for each mutant

Although PARPi are showing considerable promise as the first of a new generation of synthetic lethal therapies, resistance is a major issue^15, 16^. To better understand this, we carried out a genome-wide CRISPR-Cas9 mutagenesis screen (encompassing 87 897 single guide RNAs (sgRNAs)) to identify mouse embryonic stem (ES) cell mutants resistant to the potent PARPi talazoparib^5, 17^ (BMN673). We isolated and analysed 24 resistant clones (Methods, Fig. 1a). Nine clones harboured one of two different sgRNAs targeting *Parp1* (Table 1). *Parp1* was the only gene that was targeted by more than one different sgRNA among the resistant clones (Table 1 and Fig. 1b).

**Table 1:** Results of sequencing sgRNA PCR products from talazoparib-resistant mouse ES cell clones isolated from the CRISPR screen described in Fig. 1a

Clone	Likely cause of resistance	Clonal with	sgRNAs identified
BR1	*Parp1*	7	*Parp1* (#1), *Tmed5*
BR2	*Parp1*	—	*Parp1* (#1)
BR3		—	*Gpd2*
BR4		—	*Tbx20*, *Gpr89*, *Pde4d*, *Arhgap12*
BR5		21	*Trp53*, *Csmd1*
BR6	*Parp1*	—	*Parp1* (#2), *Apol6*
BR7	*Parp1*	12,1	*Parp1* (#1), *Abca14*, *Dtnb, Tmed5*
BR8	*Parp1* #2	—	*Parp1* (#2), *Lemd1*, *Slmo1*
BR9	*Parp1* #2	—	*Parp1* (#2)
BR10	*Parp1*	—	*Parp1* (#1), *Spock1*, *P2ry6*, *AW209491*
BR11	*Tdg*	14	*Tdg*, *Gm5597*
BR12	*Parp1* (by clonality)	7	*Abca14*, *Dtnb*
BR13	*Parp1*	—	*Parp1* (#1), *Nwd1*, *Scn10a*
BR14	*Tdg*	11	*Tdg*, *Gm5597*, *Tmed5*
BR15		—	ND
BR16		—	*Defb25*
BR17		—	ND
BR18		—	*Traf3ip1*, *Hkdc1*, *Gm4876*
BR19		—	*Dnajb11*, *Scaf8*, *Acbd3*, *Msh2*
BR20		—	ND
BR21		5	*Trp53, Csmd3*, *Chst15*
BR22		—	*Cyp2r1*, *Tmem69*
BR23		—	ND
BR24	*Tdg*	—	*Tdg*

Where clonality can be inferred from the combination of different guides observed, this is shown in column three

*ND* not determined (failure of PCR and/or sequencing)

Parp1 protein was absent in all of the PARPi-resistant clones with a *Parp1* sgRNA (Fig. 1c) with one exception (clone BR8), consistent with the observation that ablation of PARP1 expression prevents PARP1 trapping and causes PARPi resistance^5, 7^. DNA sequencing of the *Parp1* target site in clone BR8 revealed two in-frame *Parp1* mutations: c.127_129delATG and c.[127_129delATG;130T>A] (p.43delM and p.[43delM;44F>I], Fig. 1d). Both M43 and F44 residues are conserved between human and mouse PARP1 proteins and are predicted to be involved in base stacking interactions formed at the site of DNA/PARP1 interaction by the ZnFs of the PARP1 DNA-binding domain^18^. Using the PARP1 trapping assay^6^, we found that mutant Parp1 protein was not associated with the chromatin (C) fraction after talazoparib treatment, in contrast to the WT protein (Fig. 1e), suggesting that Parp1 trapping was impaired. A PARP1-GFP fusion protein with the p.[43delM;44F>I] mutation also failed to be recruited to DNA damage produced by laser microirradiation and did not produce PAR at the irradiated site, as monitored by expression of a fluorescent PAR-binding reporter, PBZ-mRuby2 (Fig. 1f; Methods; and D.B.K. and C.J.L., manuscript submitted). The magnitude of talazoparib resistance in the BR8 mutant clone was similar to that seen in *Parp1* mutants that showed complete loss of Parp1 protein expression, such as BR13 (Fig. 1g). The BR8 clone also exhibited resistance to olaparib (Fig. 1h, *p* < 0.0001, analysis of variance (ANOVA)), suggesting a drug class effect. We also noted that Parp1 mutant ES cells exhibited enhanced sensitivity to camptothecin, a topoisomerase I inhibitor (Supplementary Figure 1a), consistent with previous observations^19, 20^. Taken together, these observations suggested that loss of Parp1 DNA binding and activity caused by mutations in the ZnF domain of the protein can drive PARPi resistance.

### Parp1-independent resistance to talazoparib

We sequenced the *Parp1* sgRNA target sites in all PARPi-resistant clones but only identified *Parp1* mutations in clones with a *Parp1* sgRNA. We therefore considered alternative explanations for PARPi resistance in the other clones identified from the screen. Although *Parp1* was the only gene represented by multiple distinct sgRNAs in the PARPi-resistant cells, the hallmark of a true positive screen hit, we did identify multiple clones that carried either a sgRNA targeting *Tdg* or a sgRNA targeting *Trp53* (clones BR11, 14 and 24 for *Tdg* and BR5 and 21 for *Trp53*; Table 1). *Tdg*, which encodes thymine DNA glycosylase, was unusual in that there was only a single, rather than multiple, sgRNA design present in the screening library. We therefore designed an additional sgRNA (Supplementary Figure 1b) and generated independent *Tdg*-defective clones, each of which confirmed the PARPi resistance phenotype associated with Tdg loss (Supplementary Figure 1c, d). *Tdg* mutants exhibited WT sensitivity to camptothecin or ionising radiation (Supplementary Figure 1a, e), and displayed WT Parp1 trapping characteristics in methyl methanesulfonate (MMS)-treated cells (Supplementary Figure 1f), and thus did not phenocopy *Parp1* mutants. Tdg removes thymine, as well as some modified uracil and cytosine, residues from DNA, thus creating abasic sites^21^. It seems possible that Tdg acts upstream of Parp1 to generate substrates for Parp1 trapping, and that in its absence, a reduction in Parp1 trapping causes drug resistance, a hypothesis previously suggested^22^. However, we were unable to detect a profound general defect in Parp1 trapping in Tdg mutants (Supplementary Figure 1f) suggesting that this phenotype might be more mechanistically complex or undetectable using this approach.

Further investigation of clones with guides targeting *Trp53* (encoding the mouse homologue of p53) revealed that each had the same compound frameshift mutations and were thus likely sister clones (Supplementary Figure 1g). Sequencing of the ES cell library prior to selection revealed that all *Trp53*-targeting guides were highly enriched in the pre-PARPi-exposed population of cells (Supplementary Figure 1h). Although these clones did show significant talazoparib resistance (Supplementary Figure 1i, *p* < 0.0001, ANOVA), we would expect to have isolated more than one *Trp53* mutation event if this were a specific effect. It is therefore likely that these clones were isolated due to their high abundance in the pre-PARPi-exposed population.

### A focused CRISPR-Cas9 screen for in-frame *PARP1* mutations

**Fig. 2:**
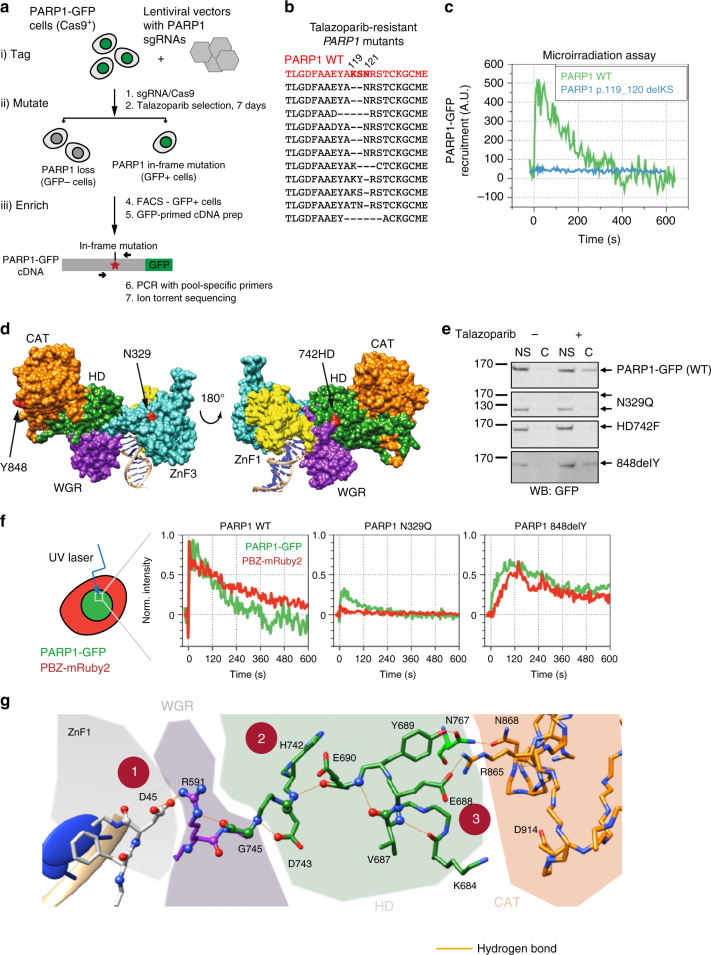
A focused CRISPR-Cas9 mutagenesis “tag, mutate and enrich” screen identifies PARP1 mutations outside the DNA-binding domain that cause PARP inhibitor resistance. **a** Experimental “tag, mutate and enrich” scheme to isolate missense and in-frame *PARP1* mutants associated with PARP inhibitor resistance. **b** Translated alignment of PARP1 amino-acid mutations identified in talazoparib-resistant cells isolated from lentiviral sgRNA pool 1 (designed to target ZnF1 and ZnF2 domains) from the screen shown in **a**. By comparing the multiple different mutations isolated from pool 1, a PARP1 p.119_120delKS minimal mutation associated with resistance was identified. Some insertions and larger deletions are omitted for clarity (see Supplementary Data 2). **c** PARP1 p.119_120delKS mutation abolishes recruitment of a PARP1-GFP fusion to sites of microirradiated DNA damage. CAL51 *PARP1*^*–/–*^ cells were transfected with a wild-type PARP1-GFP cDNA construct or a PARP1 p.119_120delKS-GFP fusion cDNA expression construct and then exposed to localised ionising radiation (a microirradiated spot) as in **f**. Time course of PARP1-GFP signal at microirradiated site is shown. **d** Location of three mutations associated with PARPi resistance on a model of the PARP1 DNA structure^12^. **e** PARP1-N329Q and HD742F mutations ablate PARP1 trapping, while 848delY partially reduces trapping. Western blot from PARP1 trapping assay for three PARP1-GFP mutants and wild-type PARP1-GFP is shown. MMS-treated cells were lysed and fractionated into nuclear soluble (NS) and chromatin (C) fractions as described in Methods. Blot was probed with an anti-GFP antibody. **f** PARP1-848delY mutation alters PARP1 localisation kinetics at sites of DNA damage. Microirradiation and PAR synthesis phenotypes of N329Q and 848delY PARP1 mutants. Green—PARP1-GFP signal, red—PAR sensor (PBZ-mRuby2). Note lower recruitment relative to wild type of both mutants, but retention of PAR synthesis in 848delY. **g** Model of intramolecular communication between the DNA-binding ZnF1 domain and the catalytic (CAT) domain based on mutants identified from screens. (1) 45delD mutation in first zinc finger, (2) HD742F mutation shown in **d**, (3) E688 (Supplementary Data 2)

Our CRISPR screens highlighted a key feature of CRISPR mutagenesis—the ability to cause and easily identify subtle mutations as well as null mutations, such as the p.43delM mutation in the BR8 clone (Fig. 1d). To study such mutations in more detail and to identify which regions of PARP1 are required for PARPi cytotoxicity, we designed an experimental approach to directly select PARPi-resistant cells with CRISPR-Cas9-induced mutations that preserved the native PARP1 reading frame (e.g., in-frame insertion/deletion mutations, rather than frameshift mutations that may cause production of truncated protein). This approach, which we term “tag-mutate-enrich” (Fig. 2a), used HeLa cells with a BAC transgene containing *PARP1* gene fused to green fluorescent protein (GFP) coding sequence (encoding a PARP1 protein with a C-terminal GFP fusion). The GFP tag allowed us to enrich for cells with in-frame PARP1 protein expression by fluorescence-activated cell sorting (FACS) isolating the GFP-positive fraction. *PARP1-GFP* cells were mutated with a focused sgRNA library of 29 guide RNAs targeting *PARP1* (Supplementary Data 1); PARPi-resistant cells were then selected via talazoparib exposure and the GFP-positive fraction isolated (Fig. 2a). The sgRNA library was introduced as six different lentiviral pools, grouped by reverse transcription-PCR (RT-PCR) product later used for genotyping. These products were amplified from GFP cDNA and sequenced using the Ion Torrent PGM platform (Fig. 2a). For all but one sgRNA pool we saw a significant enrichment of in-frame mutations in *PARP1* in the PARPi-resistant population (compared to a null hypothesis of 1/3 in-frame mutations; pools 1, 2, 4, 5 and 6: *p* < 10^−15^, binomial test with alternative hypothesis *p*(success) > 1/3, 95% confidence interval (CI) 0.62–1.00. Pool 3, 95% CI 0.24–1.00, *p* = 1; Supplementary Figure 2a). Most of these in-frame mutations were close to a CRISPR target site (95% of indels, represented by 70% of total reads, within 5 bp of a predicted target site, *p* < 1 × 10^–5^, based on Monte Carlo simulation of a randomly occurring mutation, Supplementary Figure 2b). By translating and aligning in-frame reads from PGM sequencing, we identified candidate PARP1 amino-acid residues associated with resistance. For example, most reads from the PARPi-resistant population generated from sgRNA pool 1 exhibited deletion of nucleotides encoding amino-acid residues K119 and S120 (Fig. 2b and Supplementary Figure 2a), which are both DNA-contacting residues within the second ZnF domain of PARP1^18^. We also observed numerous other alleles with deletions or insertions affecting this region (Fig. 2b and Supplementary Data 2 and 3). Our subsequent microirradiation assay analysis demonstrated that a p.119_120delKS mutation abolished PARP1-GFP recruitment to the sites of DNA damage (Fig. 2c), suggesting that mutations in residues K119 and S120 might cause PARPi resistance by altering PARP1 DNA-binding properties.

### Mutations outside the ZnF domains cause PARPi resistance

The focused sgRNA screen also identified three *PARP1* mutations in residues not known to be directly involved in DNA binding: p.329N>Q (N329Q); p.742-743HD>F (HD742F); and p.848delY (848delY, Fig. 2d and Supplementary Data 2 and 3). N329 sits within the third ZnF of PARP1, but is not predicted to form part of the DNA-binding interface. HD742F is located within the HD, a regulatory region shown to be important for PARP activation^11, 12^. Y848 is part of a solvent-exposed helix of the catalytic domain (Fig. 2d). In cell-free assays using recombinant PARP1 proteins, we found the talazoparib IC_50_ to be similar for mutant and WT PARP1 proteins (Supplementary Figure 2c, d), suggesting that the PARPi resistance phenotypes were not caused by differences in the ability of PARPi to inhibit the catalytic activity of mutant proteins. PARP1 trapping assays in CAL51* PARP1*^–/–^ cells transfected with mutant or WT PARP1-GFP cDNA expression constructs suggested that N329Q and HD742F mutant proteins were not trapped by PARPi (Fig. 2e), explaining the PARPi-resistant phenotypes associated with these mutations. In contrast, PARP1-848delY-GFP was trapped in the chromatin fraction by talazoparib, but to a lesser extent than WT PARP1-GFP (Fig. 2e). Laser microirradiation assays confirmed this partial PARP1 trapping phenotype with the PARP1-848delY-GFP protein; whilst WT PARP1-GFP was rapidly recruited to the site of microirradiation (peak ≈ 4 s) and induced PAR formation in a similar time frame (Fig. 2f), the extent of PARP1-848delY-GFP recruitment to the site of microirradiation was reduced, as was PARylation (Fig. 2f). As a negative control, PARP1-N329Q-GFP exhibited limited recruitment to damaged DNA (Fig. 2f). The addition of talazoparib delayed PARP1-848delY-GFP dissociation from microirradiated sites, but to a lesser extent than for WT PARP1-GFP (Supplementary Figure 2e, f, *p* = 5 × 10^−7^, *t*-test). PARP1-848delY-GFP also dissociated faster from microirradiated sites, being absent from microirradiated regions 30 min after microirradiation; WT PARP1-GFP still showed 40% of maximal trapping at this timepoint (Supplementary Figure 2f, *p* = 5.5 × 10^−3^, *t*-test). These data therefore suggested that even the relatively subtle defect in trapping in the 848delY mutant (Fig. 2e, f) might be sufficient to cause PARPi resistance.

We mapped the residues affected by the mutations that we observed onto a previously described crystal structure of PARP1-ZnF domains 1 and 2 together with the WGR, regulatory and catalytic domains^13^. We found that several of the PARPi resistance-causing mutations affected amino-acid residues (D45, H742, D743 and E688) involved in hydrogen-bonding interactions that bridge the DNA-binding domain and the catalytic domain (Fig. 2g), suggesting that these might control inter-domain interactions that mediate PARP1 trapping; similar inter-domain domain interactions link DNA binding to activation of PARP1 catalytic activity^11–13^. Taken together, these data established that mutations outside of the DNA-binding domain can cause PARPi resistance, likely by impairing PARP1 trapping.

### *PARP1* mutations cause PARPi resistance in *BRCA1* mutant cells

We next assessed whether *PARP1* mutations could cause PARPi resistance in a clinically relevant setting, such as in *BRCA1* mutant tumour cells. Complete loss of both *PARP1* and *BRCA1* is expected to be synthetic lethal^1, 2^. However, a growing body of evidence suggests that many pathogenic *BRCA1* mutations may not result in complete loss of function. For example, SUM149 (also known as SUM149PT) triple-negative breast tumour cells possess a commonly occurring hypomorphic *BRCA1* exon 11 c.2288delT frameshift mutation and loss of the WT *BRCA1* allele^23^. As a consequence, SUM149 cells do not express the full-length BRCA1 p220 protein, but do express a hypomorphic splice variant of BRCA1, Δ11b, which excludes the c.2288delT premature truncating mutation (along with most of the exon 11 coding sequence), but has some residual function^24, 25^. We and others have previously confirmed that the *BRCA1* mutation in SUM149 cells causes sensitivity to PARPi by demonstrating that genetic reversion of the *BRCA1* mutation in SUM149 cells via CRISPR-Cas9 mutagenesis imparts PARPi resistance^25, 26^.

**Fig. 3:**
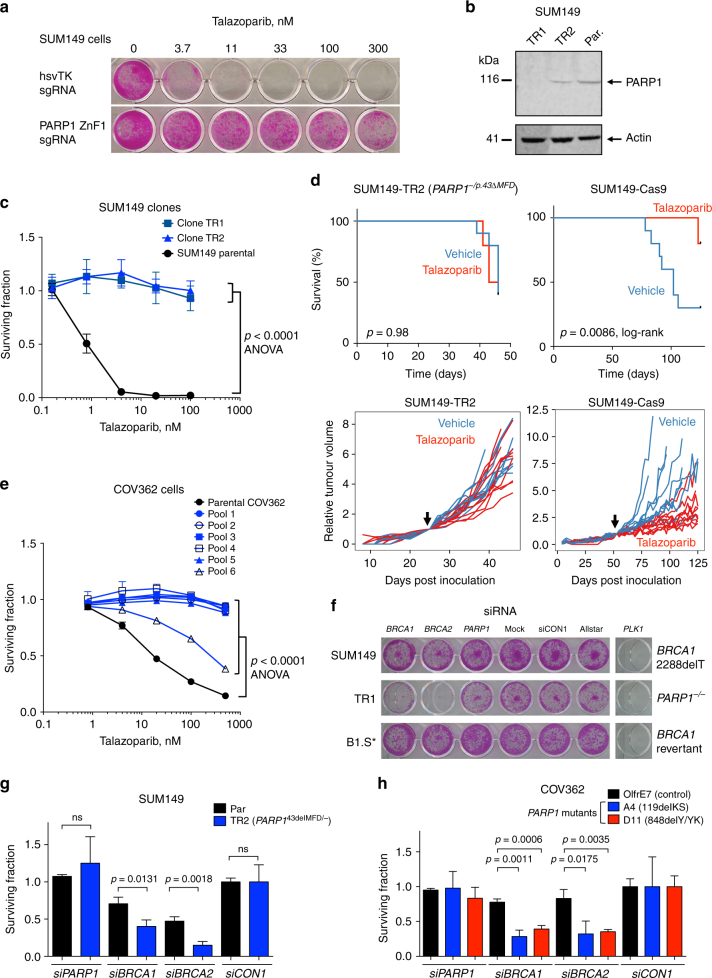
*PARP1* mediates PARPi sensitivity in *BRCA1* mutant cell lines. **a** Cas9-expressing *BRCA1* mutant SUM149 cells were transduced with PARP1-ZnF or hsvTK (non-targeting control) lentiviral sgRNA particles, selected in puromycin and exposed to the indicated talazoparib concentrations for 7 days. Representative SRB-stained 24-well plate image is shown. **b** Western blot showing PARP1 expression in talazoparib-resistant SUM149 clones TR1 and TR2. Par, parental SUM149 cells. TR2 has an in-frame PARP1-p.43_45delMFD mutation. **c** SUM149 clones TR1 and TR2 are highly talazoparib-resistant. Plot shows survival relative to DMSO-exposed cells after exposure to the indicated concentration of talazoparib for 7 days. Mean of five replicates plotted, error bars show SD. **d** PARP1 mutant SUM149-TR2 xenografts do not respond to talazoparib treatment, whereas PARP1 wild-type SUM149 xenografts do. Top panels show Kaplan–Meier survival curves, with a tumour volume of 1000 mm^3^ (after which mice were sacrificed) used as a surrogate end point. Lower panels show volume of individual tumours, normalised according to tumour volume on the day of randomisation (indicated by the arrow). **e** Pools of *PARP1* guides cause talazoparib resistance in the *BRCA1* mutant ovarian cancer cell line COV362. Cas9-expressing COV362 cells were transduced with the indicated lentiviral guide pool, selected in puromycin and assayed as in **c**. **f** Silencing of residual *BRCA1* function, or *BRCA2*, induces synthetic lethality with *PARP1* genetic loss in SUM149 cells. B1.S* is a SUM149 derivative with a secondary *BRCA1* mutation that confers PARP inhibitor resistance. SRB-stained 24-well plate image is shown, 7 days after siRNA transfection. **g** Colony forming assay using SUM149-Cas9 parental cells and the talazoparib-resistant daughter clone, TR2. Cells were transfected as in **f**, plated on 6-well plates and colonies stained and counted 2 weeks later. siRNA targeting *BRCA1* or *BRCA2* significantly reduces survival in *PARP1* mutant cells (TR2) compared to the parental SUM149 line (*t*-test). **h** *BRCA1* and *BRCA2* silencing is also synthetically lethal in COV362 *PARP1* mutant clones A4 (p.119_120delKS) and D1 (p.848delY/YK). Colony formation assay as in **g**, *p* values for *t*-test shown. Mean of three replicates plotted, error bars show SD

We carried out a genome-wide PARPi resistance CRISPR-Cas9 screen in SUM149 cells, using a previously validated sgRNA library^27^, similar to our earlier screen in mouse ES cells. Out of 12 talazoparib-resistant clones analysed, 8 possessed one of three *PARP1* sgRNAs (Table 2), suggesting that talazoparib cytotoxicity is mediated by PARP1 even in this *BRCA1* mutant cell line. To confirm this observation, we infected Cas9-expressing SUM149 cells with a lentiviral sgRNA vector targeting the *PARP1* coding sequence homologous to the p.43/44 mutation site in the PARPi-resistant mouse ES cell BR8 clone we identified earlier (Fig. 1d). This sgRNA induced talazoparib resistance (Fig. 3a). We subcloned two daughter clones from the PARPi-resistant SUM149 population, TR1 and TR2; TR1 had four different *PARP1* alleles with frameshift mutations and lacked PARP1 protein expression, whilst TR2 expressed apparently full-length PARP1 protein (Fig. 3b and Supplementary Data 4). TR2 possessed three different PARP1 alleles with frameshift mutations (Table 3 and Supplementary Data 4) but also a p.43_45delMFD in-frame deletion, similar to the p.43/44 mutations identified in PARPi-resistant BR8 mouse ES cells (Fig. 1d). This supported the hypothesis that the p.43delM mutation identified in the mouse BR8 clone (Fig. 1d) was likely to be the cause of PARPi resistance, as opposed to a passenger effect that co-occurred in a clone with a non-PARP1-mediated mechanism of resistance. No *PARP1* WT DNA-sequencing reads were identified among subcloned *PARP1* PCR products from either TR1 or TR2 (Supplementary Data 4). The allele frequencies of the *PARP1* mutations in TR1 and TR2 suggested that there were five copies of the *PARP1* locus in SUM149 cells, confirmed by single-nucleotide polymorphism genotyping of SUM149 (Supplementary Data 4; A. Grigoriadis, personal communication). Both clones remained talazoparib-resistant after culture in the absence of PARPi, demonstrating that this was a stably acquired phenotype (Fig. 3c, ANOVA, *p* < 0.0001). We did not find evidence for other known mechanisms of PARPi resistance in TR1 and TR2, such as reversion of full-length BRCA1 expression^28^ (Supplementary Figure 3a), 53BP1 or REV7 (MAD2L2) loss^29^ (Supplementary Figure 3b, c), or an increase in DNA damage-induced nuclear RAD51 foci (Supplementary Figure 3d). This suggested that PARP1 mutation or loss could not only be tolerated in SUM149 cells but also caused PARPi resistance.

**Table 2:** sgRNA sequences identified in talazoparib-resistant SUM149-Cas9 cells mutagenized with a genome-wide CRISPR library

sgRNA	Colonies
PARP1_ex16	5
PARP1_ex19	1
PARP1_ex22	1
PARP1_ex19, MCF2L_ex2	1
PARP1_ex22, JPH4_ex3	1
RABGAP1_ex8	1
ND (>2 guides)	2
	12

**Table 3:** *PARP1* mutations identified in talazoparib-resistant SUM149 and COV362 clones, determined by Sanger sequencing

Clone	sgRNA	*PARP1* mutation(s)
SUM149 TR1	ZnF 1	Frameshift mutations: c.130_133delTTTG (p.[44delF;fs49*]), c.130_131delTT (p.44F>*), c.129_130delGT (p.43M>I*), c.121-4_130_delCTAGTCGCCCATGTT [splice acceptor]
SUM149 TR2	ZnF 1	p.43delMFD and frameshift mutations: c.130_133delTTTG (p.[44delF;fs49*]), c.129_130insC (p.44F>L*), c.129delG (p.43M>fs49*).
COV362 A4	Pool 2	p.119delKS
COV362 D1	Pool 5	p.848delY/848delYK
COV362 D4	Pool 5	p.848delYKPF
COV362 D8	Pool 5	Wild type
COV362 D9	Pool 5	p.848delY/848delYK
COV362 D11	Pool 5	p.848delY/848delYK

See also Supplementary Data 4 for more detail regarding SUM149 clones

*fs* frameshift resulting in the indicated number of different amino-acid residues

*Stop codon

By generating a series of isogenic SUM149 daughter clones with different mechanisms of PARPi resistance (*PARP1* mutation, *BRCA1* reversion, deleterious *TP53BP1* or REV7 (*MAD2L2*) mutation^28–32^; Supplementary Figure 4a), we also found that SUM149 cells with *PARP1* mutations exhibited a comparable, if not greater, level of PARPi resistance than previously identified mechanisms of resistance (Supplementary Figure 4b, c; *p* < 0.0001, ANOVA compared to parental cells for all mutants). *PARP1* mutant SUM149 cells had the most profound PARPi-resistant phenotype seen amongst 38 molecularly diverse breast tumour cell lines (Supplementary Figure 5). We also found that *PARP1* mutant SUM149 cells did not exhibit cross-resistance to cisplatin that might be expected if BRCA1 function had been restored in these cells^26^ (Supplementary Figure 6a); in comparison, 53BP1 mutant clones had an intermediate level of resistance to cisplatin (*p* < 0.0001, ANOVA), as previously described^29^, as did clones with a *BRCA1* reversion, whilst *REV7* mutants exhibited enhanced sensitivity to cisplatin (*p* < 0.0001, ANOVA), a possible consequence of losing REV7’s role in translesion synthesis^33^ (Supplementary Figure 6a). *PARP1* mutation in SUM149 cells enhanced the existing topoisomerase I inhibitor sensitivity caused by *BRCA1* mutation (Supplementary Figure  6b–d, *p* < 0.0001, ANOVA), consistent with the enhanced camptothecin sensitivity caused by *Parp1* mutation in mouse ES cells (Supplementary Figure 1a) and previous observations^19, 20^. These observations suggested that *PARP1* mutation in *BRCA1* mutant tumour cells caused a similar extent of PARPi resistance as for known mechanisms of resistance, but had differing effects on platinum sensitivity.

To assess whether the PARPi-resistant phenotype caused by PARP1 mutation might also operate in vivo, we injected SUM149-Cas9 and PARP1 mutant SUM149-TR2 cells subcutaneously in BALB/c nude mice. Once tumours had established, we randomised mice into one of two treatment cohorts, one treated with the clinical PARPi talazoparib and the other with the drug vehicle alone. Whilst talazoparib treatment delayed the growth of SUM149-Cas9 xenografts and extended the survival of xenograft-bearing mice, compared to vehicle treatment (*p* = 0.0086, log-rank test, Fig. 3d), it did not have any detectable effect in mice bearing *PARP1* mutant SUM149-TR2 xenografts (*p* = 0.98, log-rank test).

### Residual *BRCA1* function supports *PARP1* loss in tumour cells

We also found that *PARP1* sgRNA caused PARPi resistance in *BRCA1* mutant COV362 ovarian tumour cells^34^ (*BRCA1* c.2611fs [exon 11] and c.4095+1G>T [exon 11 splice donor]), suggesting that these observations were not private to SUM149 cells. COV362 cells were infected with six different pools of lentiviral *PARP1* sgRNAs as used in the HeLa screen above (Fig. 2) and selected in talazoparib. We found profound talazoparib resistance in all sgRNA lentivirus-infected populations, except those infected with *PARP1* sgRNA pool six, where resistance was less-pronounced, although still significant (Fig. 3e; *p* < 0.0001 in each case, ANOVA compared to parental COV362-Cas9 cells). We also isolated a number of daughter clones from the PARPi-resistant COV362 populations. Some of these clones had lost PARP1 expression while others had in-frame indels in *PARP1*, including a clone with a p.119delKS mutation and three independently derived clones all with p.848delY/p.848_849delYK compound mutations (referred to as p.848delY/YK, Supplementary Figure 7a and Table 3). All clones with *PARP1* mutations remained resistant to talazoparib after culture without PARPi selection during subcloning and expansion (Supplementary Figure 7b, c). Interestingly, COV362 clones isolated with Y848 mutations at the endogenous *PARP1* locus (p.848delY/delYK) showed a less-pronounced resistance phenotype compared to complete null clones, although the extent of resistance was still significant compared to WT cells (Supplementary Figure 7b, c, d; *p* < 0.0001, ANOVA). These clones also showed some residual PARylation activity in agreement with the microirradiation phenotype observed earlier (Supplementary Figure 7e and Fig. 2f), suggesting an intermediate level of PARPi resistance could be caused by a partial PARP1 trapping defect.

We also attempted to isolate *PARP1* mutants from MDA-MB-436 cells, which have a *BRCA1* c.5396+1G>A mutation in the splice donor site of exon 20. This mutation results in a truncated BRCA1 protein lacking the BRCT region required for HR^23, 35^. We were unable to isolate long-term talazoparib-resistant cells from this cell line after transfection with PARP1 sgRNA-expressing lentiviral pools. Analysis of cells grown in talazoparib for a short period (Supplementary Figure 7f) showed that mutations were induced at the sgRNA target sites, but cells with PARP1 mutations did not survive in the long term (Supplementary Figure 7g). In contrast, sequencing of COV362 cells infected with the same PARP1 sgRNA pools revealed that several pools of talazoparib-resistant cells generated in the same way had mutagenized all *PARP1* alleles in all cells, indicated by very low or absent WT reads (Fig. 3e and Supplementary Figure 7g). This suggested that mutation of all *PARP1* alleles was not tolerated in MDA-MB-436 cells, in contrast to SUM149 or COV362 cells.

We therefore considered it possible that the residual *BRCA1* function in SUM149 and COV362 might allow these cells to tolerate *PARP1* mutations. To address this, we inhibited the residual BRCA1 function in SUM149 and COV362 cells with siRNA targeting *BRCA1* target sites outside exon 11; doing the same in SUM149/COV362 *PARP1* mutants isolated earlier demonstrated that *BRCA1* siRNA had a more profound cell inhibitory effect in *PARP1* mutant COV362 and SUM149 cells, than in *PARP1* WT parental cells (Fig. 3f–h, *p* < 0.05 for all cases compared to parental cells, *t*-test). This suggested that some residual BRCA1 function might exist in these cells and is required for cell survival in the face of *PARP1* mutation. *BRCA2* siRNA also selectively targeted *PARP1* mutant COV362 and SUM149 cells, suggesting some requirement for BRCA2 function in these cells despite the *BRCA1* mutation (Fig. 3f–h, *p* < 0.05, *t*-test). Taken together, these observations suggested that *PARP1* loss might be tolerated in COV362 and SUM149 cells due to some residual *BRCA1-*dependent function. Exon 11 frameshift mutations comprise approximately 30% of pathogenic *BRCA1* mutations identified to date^25, 36–39^ and thus *PARP1* mutation and/or loss could be a clinically relevant mechanism of PARPi resistance in this specific context.

### A dense CRISPR screen for functional annotation of PARP1

**Fig. 4:**
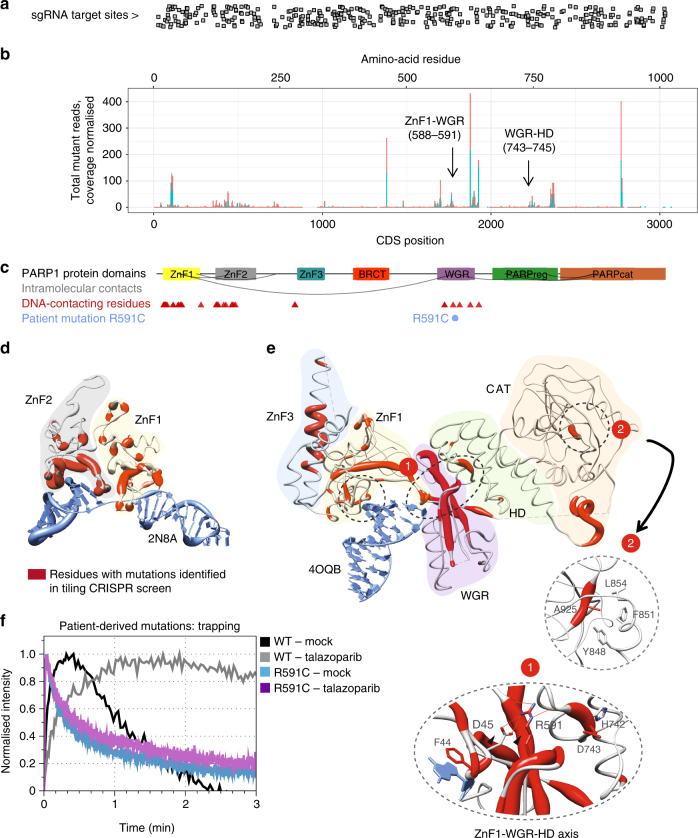
A high-density tiling CRISPR-Cas9 screen enables functional annotation of a *PARP1* mutation observed in an olaparib-resistant patient. **a** Positions of sgRNA target sites in the dense PARP1 tiling library mapped onto the PARP1 coding DNA sequence shown in **b**. **b** Positions of mutations identified in the dense *PARP1* tiling screen. Number of mutant reads for in-frame mutant alleles affecting each base is shown on the *y*-axis normalised to per-base coverage (mutant reads/total coverage × 1000). Only sites with coverage >500 reads are shown. The experiment was repeated in duplicate from two independently tagged SUM149 PARP1-GFP cell lines (clone 5 and 8, shown in red and blue). Arrows highlight the mutations observed at inter-domain contacts as shown in **e**. **c** Positions of protein domains mapped onto the PARP1 protein sequence. Red triangles indicate DNA–protein contacts based on crystal structures, blue circles show the location of the patient mutation identified in this study. Grey arcs represent key inter-domain contacts as shown in **e** and Fig. 2g. **d** Ribbon plot of ZnF1 and ZnF2 bound to a single-strand break (PDB: 2N8A). Ribbons are coloured red by residue based on the frequency of mutations observed in the tiling screen affecting that residue as shown in **b**. The thickness of the ribbon is proportional to the frequency of mutations observed affecting that residue. **e** Ribbon plot of the PARP1-DSB crystal structure (PDB: 4OQB) highlighting the clustering of mutations along the hydrogen-bonding axis postulated in Fig. 2g and the A925 residue that abuts Y848 (inset 2). Regions marked 1 and 2 are magnified. **f** Analysis of trapping for the R591C patient mutation by microirradiation with or without talazoparib as shown. Although the R591C mutant can bind DNA at sites of damage (blue line), it is not trapped in the presence of talazoparib (purple line)

To investigate which functions of PARP1 were important for cytotoxicity of talazoparib in *BRCA1* mutant cells, we designed a new tiling CRISPR library, comprising 489 guides, designed to give the densest possible coverage of *PARP1* mutations (Supplementary Data 5 and Fig. 4a) and carried out a tiling screen in SUM149 cells in which the endogenous *PARP1* locus had been tagged with a C-terminal GFP coding sequence using the CRISPaint system^40^. After mutagenesis followed by talazoparib selection and sorting GFP-positive cells as before, we sequenced the *PARP1* coding sequence from GFP cDNA using a panel of 15 amplicons (Supplementary Data 6). This screen further illuminated the roles of different areas of PARP1 in PARPi toxicity. In agreement with our previous results, a large number of mutations were isolated which affected residues associated with either direct DNA binding (Fig. 4b, c; Supplementary Data 7) or the interfaces between ZnF domains (Fig. 4d). We also observed a high density of mutations associated with PARPi resistance affecting the WGR domain (Fig. 4b, e). Based on our previous screen, we hypothesised that points of contact of the WGR domain with the ZnF and HD (regulatory) domains might be important for PARPi resistance and PARP1 function and activation (Fig. 2f); in this new screen we identified several mutation clusters at these points of contact (Fig. 4b, e; Supplementary Figure 8a) supporting this hypothesis. Although we identified few mutations in the catalytic domain that caused PARPi resistance—perhaps because mutations directly disrupting the catalytic activity would cause constitutive trapping and cytotoxicity—the most frequent mutation in the catalytic domain isolated in the screen affected residue A925, which is juxtaposed with residue Y848 in the three-dimensional structure of PARP1, further suggesting an important role for this region of the catalytic domain in PARPi cytotoxicity (Fig. 4e, inset 2). The relative lack of mutations in the BRCT domain, despite ample sequencing and sgRNA coverage in this area (Fig. 4c and Supplementary Figure 8b) might indicate that this region is dispensable for PARPi cytotoxicity and trapping.

### *PARP1* mutation observed in an olaparib-resistant patient

In parallel with these genetic studies, we identified a* PARP1* p.R591C mutation (c.1771C>T) in an ovarian cancer patient who showed de novo resistance to olaparib. The R591C mutation affects the WGR domain at the point of contact with D45 and the HD domain; our model (Fig. 2g) and previous work^11–13^ predict that this would be a critical residue for inter-domain communication in PARP1, between the DNA-binding and catalytic domains, implying that this mutant might bind DNA but would not show trapping. To investigate this, we assessed the recruitment of a PARP1-R591C-GFP fusion protein to sites of microirradiation in the presence and absence of the PARPi talazoparib. We found that whilst PARP1-R591C-GFP was recruited to sites of microirradiation, its dissociation from these sites was rapid compared to WT PARP1-GFP; furthermore, PARP1-R591C-GFP was not trapped on DNA by talazoparib, unlike WT PARP1-GFP (Fig. 4f).

## Discussion

A key observation that contributed to the trapping hypothesis of PARPi cytotoxicity is that in HR proficient cells, PARP1 itself is required for cytotoxicty^6, 7^. Our genome-wide CRISPR screen revealed that point mutations in the ZnF domains that abolish DNA binding were sufficient for this resistance, providing direct genetic evidence for the trapping hypothesis.

Our genetic screens also uncovered several clusters of mutations that suggest that regions of PARP1 outside the DNA-binding domain can influence trapping (Figs. 2d and 4b), observations that are consistent with inter-domain interactions being critical for PARP1 binding and activation^11–13^. These observations are also supported by an orthogonal approach using ethyl methyl sulfonate mutagenesis screens in haploid cells^41^. These observations add weight to the “reverse allostery” hypothesis proposed by Murai et al.^6^, which suggests that PARPi binding to the catalytic domain of PARP1 allosterically influences interactions between DNA and the N-terminal DNA-binding domains of the protein, to the extent that PARP1 becomes trapped on DNA. It is possible that the PARP1 mutations we have identified here are in amino-acid residues critical to this reverse allosteric process and therefore prevent allosteric enhancement of DNA binding upon inhibitor binding.

We also isolated a PARP1 mutant, p.848delY, which was associated with PARPi resistance despite exhibiting some residual PARP1 trapping. This reduced level of trapping might be sufficient to cause resistance—the amount of trapped PARP1 required to induce cytotoxicity is not known. Nor is it known whether all trapping, as observed by chromatin fractionation assays in the presence of alkylating agents, is equal with respect to cytotoxicity—PARP1 trapping at certain sites or lesion types may be more toxic than at others. It is possible that Tdg is a source of such lesions in ES cells, as loss of this specific glycosylase was sufficient for pronounced PARPi resistance (Supplementary Figure 1d). Alternatively, it is possible that PARP1 trapping is not, in itself, sufficient for cytotoxicity and other factors altered by the mutations are also required. For example, the Y848 residue is part of a solvent-exposed helix in PARP1 that may participate in protein–protein interactions—for example, with Timeless^42, 43^—that could be important for generating a DNA lesion that is cytotoxic. Mutations in another residue (A925) juxtaposed with Y848 in the PARP1 tertiary structure also led to PARPi resistance, possibly via a similar mechanism (Fig. 4e). It is possible that a larger trapped complex of PARP1 and interacting partner(s) is responsible for cytotoxicity, or that trapped PARP1^848delY^ is altered in such a way as to not be cytotoxic. Nevertheless, the resistance phenotype of cells with mutations affecting Y848 at the endogenous *PARP1* locus is slightly less-pronounced than a complete PARP1 knockout (Supplementary Figure 7d), supporting the hypothesis that the residual PARP1 trapping corresponds to some residual PARPi cytotoxicity. It will be interesting to study this region further to pinpoint the cause of the reduced cytotoxicity.

Our experiments also showed that *PARP1* mutation can be tolerated in certain *BRCA1* mutant, PARPi-sensitive tumour cells. This suggests that PARP1 trapping still underlies the increased cytotoxicity of PARPi in these tumour cells but that some residual BRCA1 function allows these cells to tolerate PARP1 mutations (Fig. 3f–h), consistent with previous observations that some *BRCA1* exon 11 mutations do not result in complete loss of BRCA1 function^24, 25^. Since *PARP1;BRCA1* double-mutant cells show distinct drug sensitivities compared to cells that become PARPi resistant via other mechanisms, knowledge of the mechanism of resistance in patients that relapse could inform the best subsequent treatment. Importantly, we also observed a *PARP1* mutation that abolished trapping (R591C, Fig. 4f) in a patient with de novo resistance to olaparib, suggesting that such mutations can arise in patients and could potentially contribute to resistance. It also seems reasonable to think that PARPi resistance in some patients might display some level of heterogeneity, with multiple different PARPi subclones emerging with distinct mechanisms of resistance; recent advances in the genomic profiling of both solid and liquid biopsies derived from PARPi-resistant patients^44–46^ might potentially assess whether this is the case. Whether this turns out to be the case or not, our observation that PARPi-resistant cells with different mechanisms of resistance display different chemotherapy sensitivities (Supplementary Figure 6) suggests that defining the molecular features of PARPi-resistant disease biopsies might be important to determine the best course of subsequent treatment.

The “tag-mutate-enrich” approach we have used, where the tagging of genes with C-terminal GFP coding sequences (or other selectable genes) followed by the targeted mutagenesis of these genes via CRISPR-Cas9 mediated mutagenesis, allows, in principle, full-length mutants of any gene of interest associated with a selectable phenotype to be identified. This could be employed in the analysis of other resistance mutations observed in patients being treated with targeted therapies in order to annotate likely drivers and passengers of resistance.

## Methods

### Cell lines

Mouse ES cells were cultured in Knockout DMEM containing 15% fetal bovine serum (FBS) and leukaemia inhibitory factor as previously described^47^. HeLa, CAL51 (source: DSMZ), COV362 (source: ECACC) and MDA-MB-436 (source: ATCC) cells were maintained in Dulbecco’s modified Eagle’s medium (DMEM) supplemented with 10% FBS (with 10 µg/ml insulin in the case of MDA-MB-436). The HeLa cell line expressing PARP1-GFP gene from a bacterial artificial chromosome has been previously described^48^. SUM149PT cells (referred to as SUM149, source: Asterand Bioscience) were maintained in Ham’s F-12 medium supplemented with 5% FBS, 10 µg/ml insulin and 1 µg/ml hydrocortisone.

All human cell line identities were confirmed by STR typing and verified free of mycoplasma infection using Lonza MycoAlert.

### Genome-wide mouse CRISPR screen

We screened a previously described library of mouse ES cells infected with a lentiviral single guide RNA (sgRNA) library targeting 19,150 mouse genes (average five sgRNAs/gene)^49^. Two million CRISPR mutagenised cells were exposed to a normally lethal concentration (25 nM) of the clinical PARPi talazoparib^17^ (a.k.a. BMN 673; Fig. 1a) for 6 days and allowed to form colonies. Colonies were picked and expanded in 96-well plates. sgRNA sequences from resistant clones were amplified using U6-F and CRISPR-scaf-R primers. *Parp1* genotyping of resistant clones coming from the screen was done with Parp1_CRISPRseq-F and Parp1_CRISPRseq-R primers (Supplementary Data 8) using ReadyMix Taq polymerase (Sigma) and the following conditions: 94 °C for 30 s; 30 cycles of 94 °C for 10 s; 56 °C for 10 s and 72 °C for 30 s; and the final extension, 72 °C for 4 min.

### Genome-wide CRISPR screen in SUM149 cells

SUM149-Cas9 cells were generated by transduction of SUM149 cells with a Cas9-bsd lentivirus and selection in 7 µg/ml blasticidin. Cells were infected at multiplicity of infection (MOI) 0.3 with a previously published genome-wide human lentiviral CRISPR library^27^. Cells were selected with puromycin and then placed under talazoparib selection at a concentration that killed all non-infected SUM149 cells (100 nM). Twelve surviving colonies were picked and analysed for the presence of sgRNA sequences by PCR and Sanger sequencing as above.

### Genetically engineered cell lines

CAL51 *PARP1*^–/–^ cells were generated using the Edit-R Gene Engineering System (GE Dharmacon). Cells were seeded at a density of 1 × 10^5^ cells/well in 24-well plates. After 24 h, cells were transfected with 1 µg Edit-R CRISPR-Cas9 Nuclease Expression Plasmid mixed with 2.5 µl of 20 µM PARP1 T2 crRNA (GACCACGACACCCAACCGGAGUUUUAGAGCUAUGCUGUUUUG) and 2.5 µl of tracrRNA (20 µM), using Lipofectamine 3000 according to the manufacturer’s instructions (Life Technologies). Four days after transfection, cells were selected in 100 nM talazoparib for 5 days, and surviving cells FACS-sorted into 96-well plates at one cell per well in drug-free medium. After 2 weeks, the medium was changed to 100 nM talazoparib and cells kept under selection for another month. Targeted genome modifications were analysed by Sanger sequencing of PCR products cloned into pCR-TOPO-blunt (Life Technologies). Ten individual colonies were sequenced. Clone T2.4, referred to as CAL51 PARP1^–/–^, lacks PARP1 expression by western blot and has bi-allelic out-of-frame deletions at the target site: a 50 bp deletion and a single base “C” deletion, respectively.

Stable Cas9-expressing human cell lines were generated by transducing cells with a Cas9-blasticidin lentivirus^50^ and selecting cells with integrated virus by culturing in medium containing 7 µg/ml blasticidin.

SUM149 TR1 and TR2 were generated by infecting SUM149-Cas9-expressing cells with a pLentiGuide-puro^50^ vector expressing an sgRNA targeting ZnF1 (Hs_PARP1_DBD_cr) and selecting cells with 100 nM talazoparib for 7 days. Single cells were sorted using FACS and the resulting clones genotyped by PCR with Hs_PARP1DBD-genoF and Hs_PARP1DBD-genoR primers (Supplementary Data 8) using Q5 High-Fidelity DNA Polymerase (NEB) with the following conditions: 98 °C for 30 s; 30 cycles of 98 °C for 10 s; 63 °C for 10 s and 72 °C for 30 s; and the final extension, 72 °C for 2 min.

SUM149 PARP1-tagGFP2 cells were generated by CRISPaint^40^ using as a gene-specific target sgRNA sequence 5′-GCAATTTTAAGACCTCCCTG-3′. GFP-positive single-cell clones were isolated. They were genotyped with the Hs_PARP1_intron22_F and tagGFP2_R primers (98 °C for 30 s, 30 cycles of 98 °C for 10 s, 65 °C for 10 s and 72 °C for 30 s, and the final extension, 72 °C for 2 min), and the PCR product was Sanger sequenced to ensure a single in-frame integration. Clones 5 and 8 originated from independent integration events.

*Tdg* mutant mouse ES cell clones were generated from JM8A3 (C57BL/6N) cells^47^ by infection with a single lentivirus expressing a *Tdg* targeting sgRNA (Tdg_gRNA) and Cas9 (lentiCRISPR^51^). Colonies were picked and genotyped by PCR with Tdg_CR2_checkF and Tdg_CR2_checkR primers (Supplementary Data 8) using ReadyMix Taq polymerase (Sigma) and the following conditions: 94 °C for 30 s; 30 cycles of 94 °C for 10 s; 56 °C for 10 s and 72 °C for 30 s; and the final extension, 72 °C for 4 min.

### Reagents

Drugs, including olaparib and talazoparib, were purchased from Selleckchem, except temozolomide and methyl methanesulfonate (Sigma-Aldrich). Antibodies used are listed in Supplementary Data 9.

### Cell viability and clonogenic assays

The viability of cells was measured after 5 days exposure to various concentrations of drugs using the Cell Titre-Glo assay (Promega). Long-term drug exposure effects were assessed by colony formation assay after 7–10 days exposure to a drug as described previously^1^, with drug-containing medium refreshed weekly and cells stained at the end of the assay with sulforhodamine B. When plotting survival curves, the surviving fraction was calculated relative to DMSO (solvent)-exposed cells.

### PARP1 sgRNA screen in HeLa cells

In all, 29 sgRNAs were designed using the ChopChop algorithm^52^. The guides were synthesised and cloned in an array format into pLentiGuide-puro^50^ by Eurofins genomics. A single colony for each vector was used to inoculate a pooled culture for plasmid maxi prep (Qiagen). Six such pools of vectors were packaged into viruses as previously described^53^. These pooled viruses were used to infect HeLa PARP1-GFP/Cas9 cells. sgRNA-expressing cells were enriched with a 3-day puromycin selection (3 µg/ml). The cells were subsequently incubated in 1 µM talazoparib for 12 additional days. The GFP-positive population was enriched by cell sorting (BD FACSAria). Total RNA was extracted from each of the six pools. One microgram of total RNA was converted to cDNA with SuperScript III (ThermoFisher) and a GFP-specific primer (5′-CCTTGATGCCGTTCTTCTGCTTG-3′), which allowed for amplification only of the target *PARP1-GFP* transcripts. Each of the six pooled cDNAs was amplified with its corresponding Ion Torrent adapted primer pair flanking the gRNA target sites for that pool. This amplification used Q5 High-Fidelity DNA Polymerase (NEB) with the following PCR conditions: 98 °C for 30 s; 30 cycles of 98 °C for 10 s; 64 °C for 10 s and 72 °C for 30 s; and the final extension, 72 °C for 2 min. Primer sequences are provided in Supplementary Data 8.

### PARP1 dense screen in SUM149 cells

A dense CRISPR library comprising 489 guides targeting *PARP1* was synthesised (Twist Biosciences, Supplementary Data 4) and cloned into the BbsI site of pKLV5-U6gRNA5-PGKPuroBFP^27^. One million cells from each of two SUM149 PARP1-tagGFP2 clones were infected at MOI 0.3 as above and selected in puromycin. Cas9 was induced by treatment with doxycycline for 2 days after which cells were replated and 100 nM talazoparib added. cDNA was prepared using a GFP primer as above and 15 overlapping amplicons prepared by PCR with Ion Torrent adapter tailed primers for genotyping (Supplementary Data 5).

### Sequencing and analysis

Purified RT-PCR products were mixed and sequenced using the Ion Torrent PGM and a 318 chip with 850 flows. Data were converted to FASTQ format and aligned to the *PARP1* cDNA sequence (ENST00000366794) using Novoalign (Novocraft technologies). Mutations were called from the alignments using the Ensembl Variant Effect Predictor REST API^54^ (implementation: github.com/GeneFunctionTeam/bioruby-sam-mutation). Sequences were also translated and multiple alignments generated using Clustal Omega (http://www.ebi.ac.uk/Tools/msa/clustalo/). For the dense screen, coverage was calculated per base using samtools pileup (github.com/samtools, v1.5) with a maximum depth of 300,000.

### Microirradiation assays

Cells were grown in glass-bottom culture dishes (MaTek, P35G-0.170-14-C) in 10% FBS DMEM media and maintained at 37 °C and 5% CO_2_ in an incubation chamber mounted on the microscope. Imaging was carried out on Andor Revolution system, ×60 water objective with micropoint at 365 nm. Only cells with similar GFP signal intensity were measured. The background intensity (in the vicinity of the microirradiation area in the nucleus) was subtracted from that at the microirradiation point and the maximum was normalised to 1.

### Chromatin fractionation (trapping assay)

The chromatin fractionation assay for PARP trapping was based on a previously published protocol^6^. Cells were grown in six-well plates, exposed for 1–4 h to 500 nM talazoparib and 0.01% MMS and fractionated with the Subcellular Protein Fractionation kit for Cultured Cells (ThermoFisher #78840) according to the manufacturer’s recommendations. Equivalent volumes of each fraction were analysed by western blot using the PARP1 (Cell Signaling #9542, rabbit), with TOP1 (BD #556597, mouse) or Histone H3 (CST #9717, rabbit; low molecular weight region of blot probed separately) as fractionation controls. Blots were imaged using the LiCor Odyssey multicolour imaging system and secondary antibodies (Donkey anti-rabbit 680 and donkey anti-mouse 800). Uncropped scans of western blots are shown in Supplementary Figure 9.

### Protein purification

PARP1 point mutations were introduced in an isopropyl β-d-1-thiogalactopyranoside (IPTG)-inducible C1-PARP1 plasmid (expressing His-MBP-tagged human PARP1 cDNA) and transformed in Rosetta *Escherichia coli* (Novagen). Cells were grown in terrific broth at 37 °C until reaching OD_600_ 1.0, 100 mM ZnSO_4_ was added and cells further grown to OD_600_ 2.0. A unit of 0.5 mM IPTG was added and cells were shaken at 18 °C overnight. Pellets were collected by centrifugation and stored at −80 °C. Pellets were lysed (50 mM Tris-HCl (pH 7.5), 500 mM NaCl, 10 mM imidazole, 10 mM mercaptoethanol and protease inhibitors), sonicated and cleared by centrifugation and 0.45 μm filtration. The lysate was passed through a 5 ml His Trap column (GE). The column was washed and bound proteins eluted with a linear imidazole gradient. Pooled PARP1-containing fractions were dialysed overnight (50 mM Tris-HCl (pH 7.5), 250 mM NaCl and 5 mM mercaptoethanol) in the presence of MBP-TEV protease. Dialysed proteins were cleared through 0.45 μm filter and cleaved MBP and MBP-TEV were removed through another 5 ml His Trap column. Samples were loaded on a 5 ml HiTrap Heparin column (GE) and eluted with a linear salt gradient. PARP1-containing fractions were pooled, concentrated to 1 ml and injected on a HiLoad 16/600 Superdex 200 column (GE) equilibrated in 25 mM HEPES-NaOH (pH 7.5), 100 mM NaCl and 2 mM TCEP. PARP1-containing fractions were pooled, concentrated and snap-frozen in liquid N_2_.

### PARylation assay

DNA dumbbell ligand (DB4) was prepared as previously described^12^. In vitro PARylation reactions were carried out in a buffer (50 mM HEPES (pH 7.5), 150 mM NaCl, 10 mM MgCl_2_ and 1 mM dithiothreitol) containing 100 nM PARP1 protein and 1 µM DB4. The reaction was started by the addition of NAD^+^ to a 225 µM final concentration (spiked with 5 µCi/ml ^32^P-NAD^+^) and incubation at 30 °C. Aliquots were taken at various time points and the reaction was stopped by the addition of 2× SDS-polyacrylamide gel electrophoresis (SDS-PAGE) loading dye and heat denaturation. Samples were resolved on SDS-PAGE, dried and exposed on a phosphoimager plate (GE). Total radioactive intensity was quantified in the lanes between the well start and the position of PARP1.

### Xenograft experiment

Xenograft experiments were performed by Crown Bioscience UK. Female BALB/c nude mice aged 6–8 weeks were purchased from Envigo. Mice had access to irradiated G.19% extruded rodent diet from Harlan Teklad (cat# 2919) throughout study. To form xenografts 5 × 10^6^ cells were injected subcutaneously into the left flank with 1:1 (v/v) Matrigel as previously described^17^. Tissue from established tumours was pooled and passaged with 25 µl Cultrex per mouse to new hosts for the treatment study (100 mm^3^/mouse, *n* = 20/cell line). Sample size was determined based on previous experience with this model system. Mice were randomised when average tumour size reached 100 mm^3^, using the multi-task method in the Studylog software, minimising differences in tumour volume between groups. Talazoparib (0.66 mg/ml in DMSO) was diluted in diluent (10% DMAc and 6% Solutol in phosphate-buffered saline) and administered orally on a schedule of 3 days on, 4 days off. For the vehicle arm, an equivalent volume of DMSO was diluted as above and administered on the same schedule. Tumour volumes were estimated three times per week using the formula 0.5 × length × width^2^. For survival analysis, a final tumour limit of 1000 mm^3^ was used. Blinding was not used. Animal welfare for this study complies with the UK Animals Scientific Procedures Act 1986 in line with Directive 2010/63/EU of the European Parliament and the Council of 22 September 2010 on the protection of animals used for scientific purposes. All experimental data management and reporting procedures were in strict accordance with applicable Crown Bioscience UK Guidelines and Standard Operating Procedures.

### siRNA transfection

Lipofectamine RNAimax was used according to the manufacturer’s instructions (Invitrogen). Cells were transfected with Smart Pools targeting the appropriate gene (GE Dharmacon).

### Patient tumour sequencing

The *PARP1*:R591C mutation was identified in the archival tumour sample of a 69-year-old patient (without a *BRCA1* or *BRCA2* germline mutation) with platinum-resistant high-grade serous ovarian cancer enroled in a phase I trial of olaparib and durvalumab^55^ (NCT02484404). A panel of cancer susceptibility and DNA repair genes (BROCA-HR^56^) was sequenced and the *PARP1*:R591C mutation (genomic: chr1:226 564 979G>A) was identified at a variant allele frequency (VAF) of 0.33 (65/198 reads). This ovarian carcinoma also had a *TP53* mutation (p.R196*) with VAF of 0.72. The patient did not have a response to olaparib and durvalumab based on RECIST v1.1 criteria. The study has been conducted in accordance with ethical principles that have their origin in the Declaration of Helsinki and are consistent with the International Council on Harmonization guidelines on Good Clinical Practice, all applicable laws and regulatory requirements, and all conditions required by a regulatory authority and/or institutional review board. The study protocol was reviewed and approved by the Institutional Review Board of the Center for Cancer Research, National Cancer Institute. Written informed consent was obtained from all patients^55^.

### Data availability

Data and cell lines are available from the authors on request. Sequencing data are deposited at the ENA, accession number PRJEB24332.

## Electronic supplementary material

Supplementary Information

Description of Additional Supplementary Files

Supplementary Data 1

Supplementary Data 2

Supplementary Data 3

Supplementary Data 4

Supplementary Data 5

Supplementary Data 6

Supplementary Data 7

Supplementary Data 8

Supplementary Data 9
