# Supplementary material for: Genome-wide and high-density CRISPR-Cas9 screens identify point mutations in PARP1 causing PARP inhibitor resistance
Source: Nat Commun. 2018 May 10;9:1849. doi: 10.1038/s41467-018-03917-2 (PMC5945626; doi:10.1038/s41467-018-03917-2)
Supplement: Supplementary file 1 — Supplementary Information [file 41467_2018_3917_MOESM1_ESM.pdf]

Genome-wide and high-density CRISPR-Cas9 screens identify point mutations in *PARP1* causing PARP inhibitor resistance

Stephen J. Pettitt[1,2*], Dragomir B. Krastev[1,2*], Inger Brandsma[1,2], Amy Dréan[1,2], Feifei Song[1,2], Radoslav Aleksandrov[5], Maria I. Harrell[6], Malini Menon[1,2], Rachel Brough[1,2], James Campbell[1,2], Jessica Frankum[1,2], Michael Ranes[4], Helen N. Pemberton[1,2], Rumana Rafiq[1,2], Kerry Fenwick[3], Amanda Swain[3], Sebastian Guettler[4], Jung-Min Lee[7], Elizabeth M. Swisher[6], Stoyno Stoynov[5], Kosuke Yusa[8], Alan Ashworth[9#] and Christopher J. Lord[1,2#]

[1]The CRUK Gene Function Laboratory, [2]Breast Cancer Now Toby Robins Research Centre, [3]Tumour Profiling Unit and [4]Divison of Structural Biology, The Institute of Cancer Research, London, SW3 6JB, UK. [5]Institute of Molecular Biology 'Roumen Tsanev', Bulgarian Academy of Sciences, Sofia 1113, Bulgaria. [6]University of Washington School of Medicine. 1959 NE Pacific St, Seattle, WA 98195, USA. [7]Center for Cancer Research, National Cancer Institute, Bethesda, MD 20892, USA. [8]Wellcome Trust Sanger Institute, Hinxton, Cambridgeshire, CB10 1SA, UK. [9]Current address: UCSF Helen Diller Family Comprehensive Cancer Center, 1450 3rd St, San Francisco, CA 94158, USA.

[*]Equal contribution (SJP and DBK)
[#]Correspondence to: Chris.Lord@icr.ac.uk, Alan.Ashworth@ucsf.edu

Supplementary Information

Supplementary Figures 1-9

Supplementary Figure 1

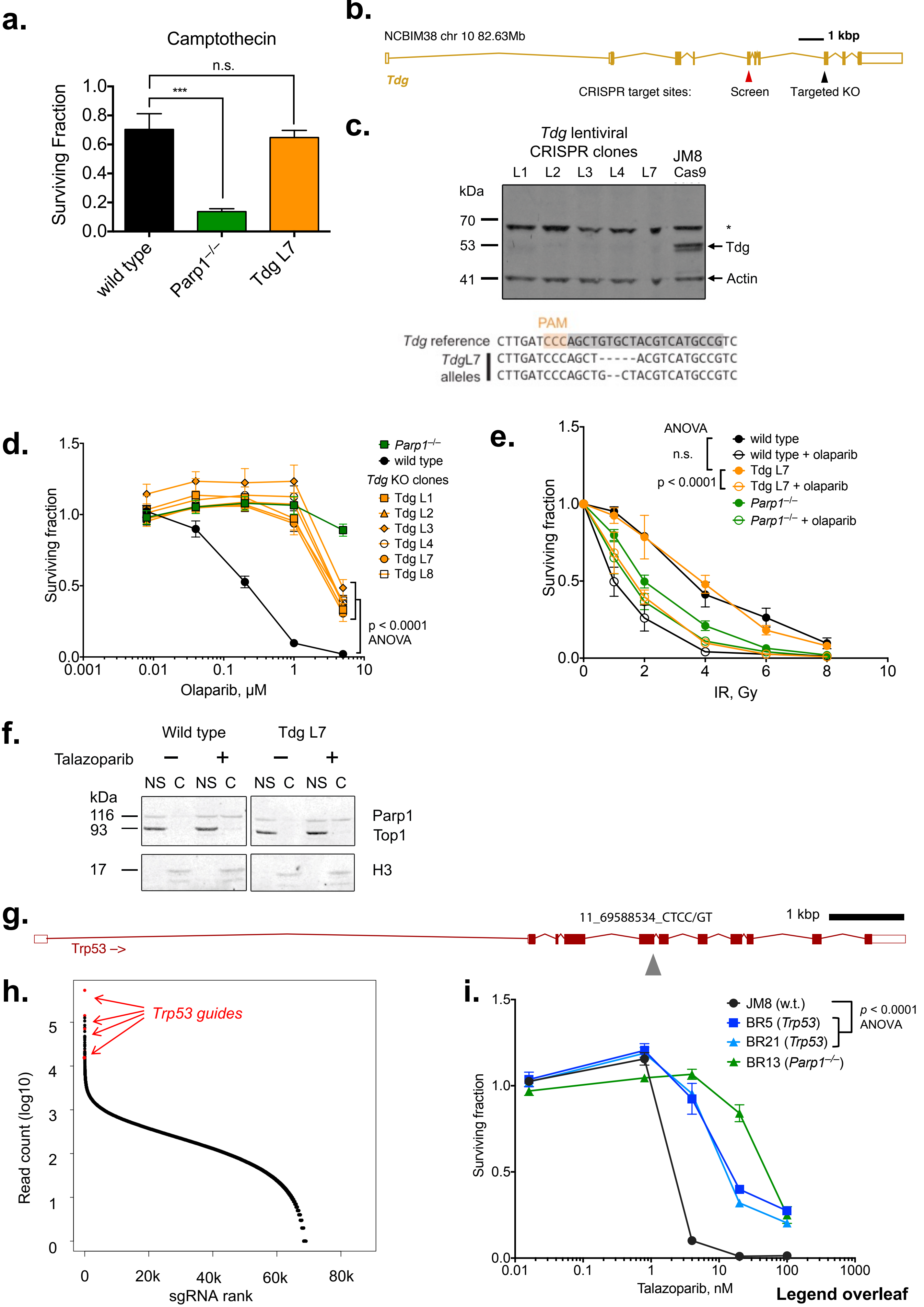

Legend overleaf

Supplementary Figure 1. Analysis of talazoparib-resistant mutants with guides targeting *Tdg* and *Trp53*. (a) *Tdg* knockout does not result in sensitivity to camptothecin, whereas *Parp1* does ($p < 0.0001$, t-test). Cells were exposed to 22 nM camptothecin for seven days and survival assayed using CellTiter Glo. Surviving fraction relative to DMSO treated cells is shown, n = 5. ns, not significant. **(b)** Location of *Tdg* sgRNA target site identified in the screen (red) and used for producing *Tdg* targeted knockout ES cells (black). **(c)** Western blot showing lack of Tdg protein expression in targeted clones. Sequencing of the mutation in TdgL7 is shown below. **(d)** *Tdg* knockout clones (orange) are resistant to olaparib. **(e)** Tdg knockout does not result in sensitivity to ionising radiation (IR), and IR sensitivity can still be potentiated by PARP inhibition in *Tdg* knockout cells, indicating that Parp remains functional. **(f)** *Tdg* knockout cells show normal levels of Parp1 trapping. Lysates from cells exposed to talazoparib as shown were fractionated and blots probed with the antibodies against the indicated proteins. All samples were treated with 0.01% MMS. NS – nuclear soluble, C – chromatin, H3 – histone H3. **(g)** Location of sgRNA target site (grey arrow) and mutation in talazoparib-resistant clones BR5 and BR21, which have *Trp53* sgRNA. **(h)** *Trp53* sgRNA vectors are highly enriched in the starting population for the screen. sgRNA coding sequences were amplified from DNA from the mouse ES cell library used for the screen and sequenced. Read counts for each sgRNA identified are plotted in rank order, with *Trp53* targeting guides shown in red. **(i)** *Trp53* mutants isolated in the screen (BR5, BR21, blue) are resistant to talazoparib compared to w.t. cells. In panels (a), (d), (e) and (i), the mean of five replicates is plotted with error bars showing s.d..

Supplementary Figure 2

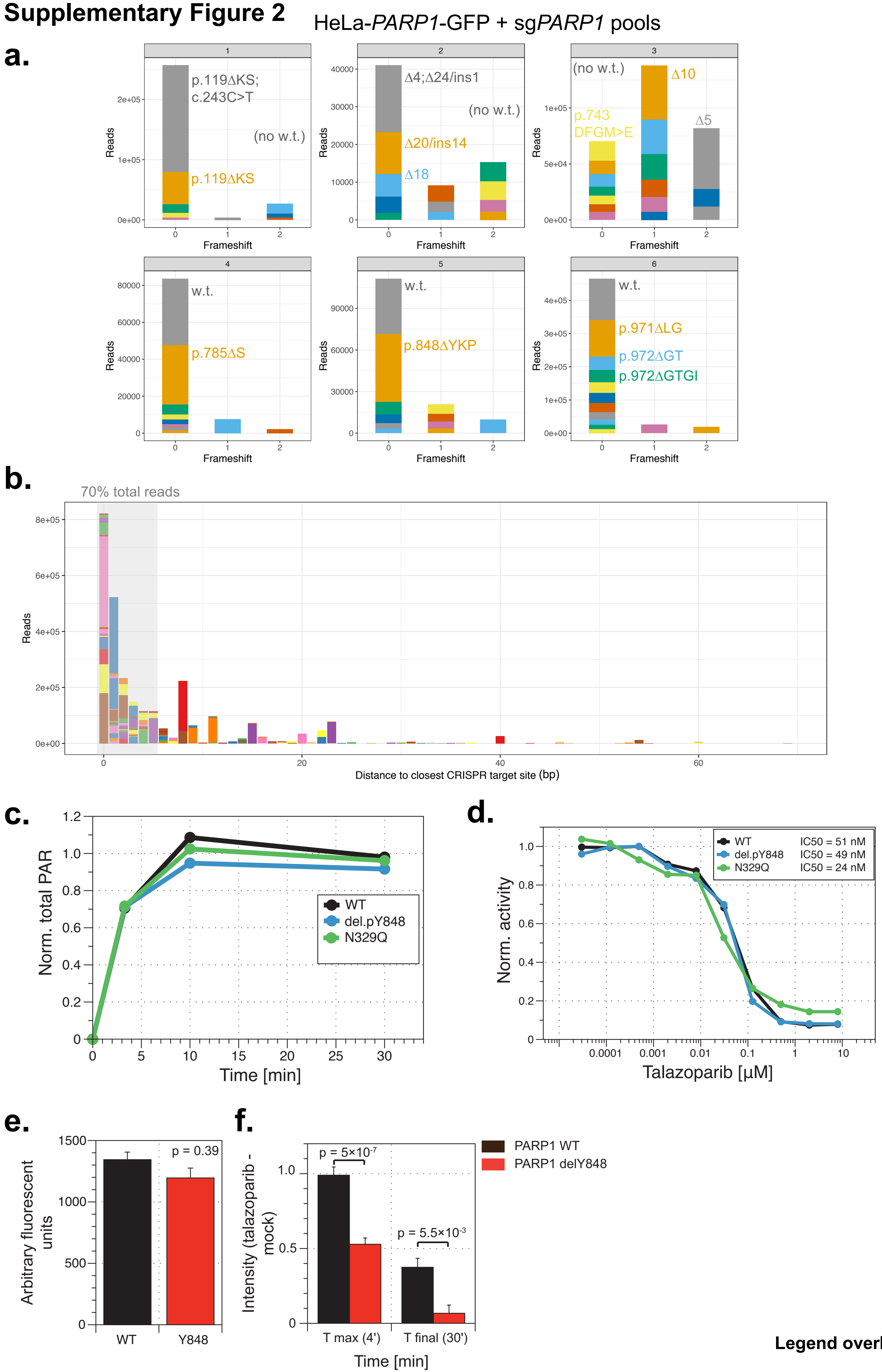

Legend overleaf

Supplementary Figure 2. Isolation of in frame PARP1 mutations using a focused CRISPR screen. (a) Allele spectrum determined from Ion Torrent sequencing of talazoparib resistant populations from lentiviral transductions of HeLa reporter cells with *PARP1* sgRNA pools 1-6. Different colours represent different mutant alleles identified (colours are repeated), with heights indicating the number of reads observed. Only alleles with read numbers > 10% of the maximum read count for each sample are plotted. Major alleles in each pool are indicated in their corresponding colour. Frameshift 0 = native reading frame. **(b)** Barchart showing distance to closest CRISPR target site and number of reads for each in-frame allele identified in Ion Torrent sequencing data. Different colours represent different alleles. **(c)** PARP activity assay for mutants identified from the screen. Formation of PAR polymer by the indicated purified PARP1 proteins in the presence of DNA was monitored by incorporation of radiolabelled NAD into high molecular weight material. **(d)** Dose-response curve of inhibition of PARP activity by talazoparib for the two mutants that retain *in vitro* PARP activity. PARP activity assay was carried at as in A with the addition of talazoparib at the indicated concentrations. PAR formation was normalised to DMSO treated control for each protein. **(e)** Expression of PARP1-p.848delY-GFP in the microirradiation assay. GFP signal for wild type and mutant fusion proteins is shown prior to irradiation. **(f)** Lower maximal trapping of PARP1-p.848delY-GFP (4 minute timepoint) and faster dissociation (30 minute timepoint) compared to wild type. Average GFP intensity in the presence of talazoparib was corrected by subtracting average GFP intensity at the same timepoint for mock treated cells to give a measure of talazoparib-dependent trapping of PARP1. P-values were calculated using a t-test.

Supplementary Figure 3
PARP1 mediates PARPi cytotoxicity in *BRCA1* mutant cell lines.

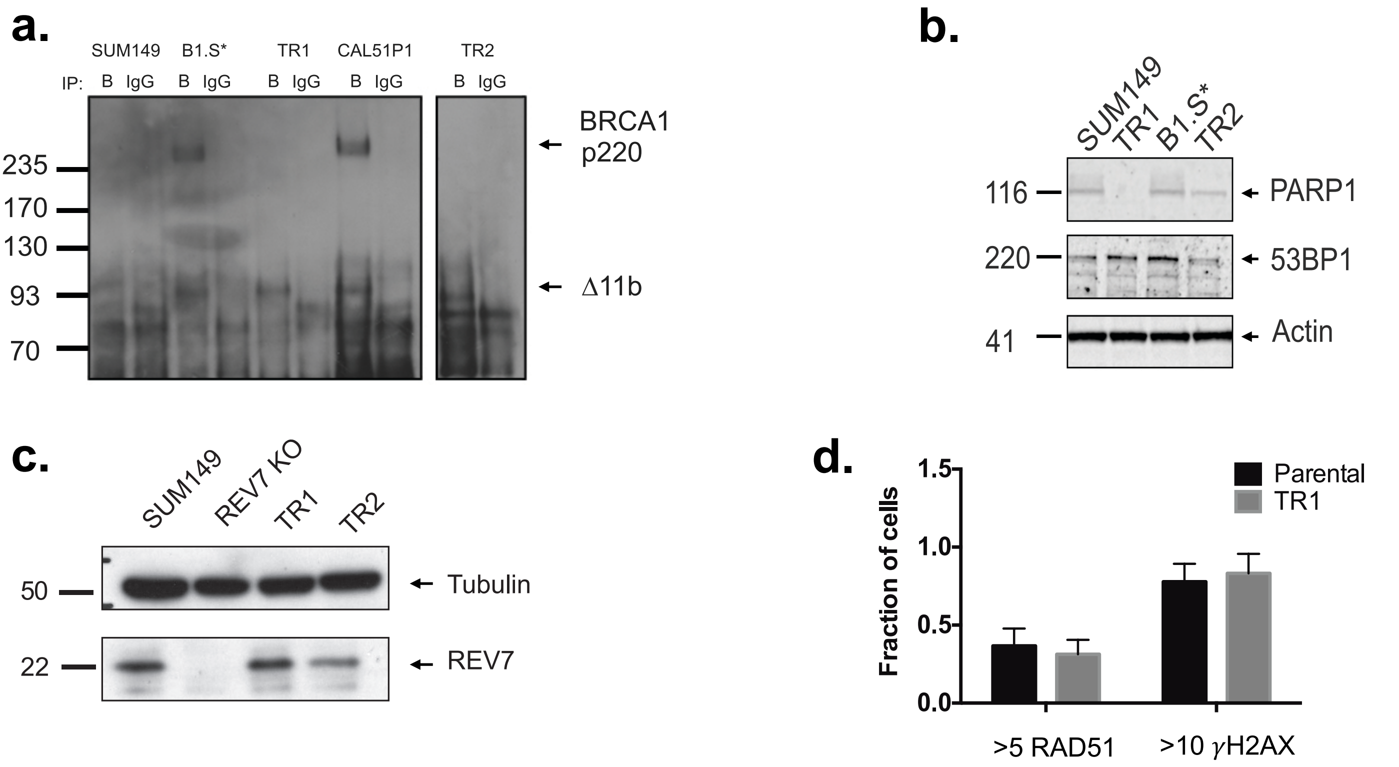

Supplementary Figure 3. PARP1 mediates PARPi cytotoxicity in *BRCA1* mutant cell lines. (a) Talazoparib-resistant cell lines SUM149-TR1 and –TR2 do not re-express full length BRCA1 protein. BRCA1 IP-western was carried out as previously described[24]. SUM149-B1.S* is a cell line in which the *BRCA1* frameshift mutation has been reverted via CRISPR mutagenesis[26]. **(b)** Talazoparib-resistant cell lines have not lost 53BP1 expression. Input lysates from (B) were probed with the indicated antibodies. **(c)** Talazoparib-resistant SUM149 cells continue to express REV7. A SUM149 line with a CRISPR generated mutation in REV7 (Supplementary Figure 4a) is shown as a control. **(d)** SUM149 talazoparib resistant cells (TR1) do not have increased levels of RAD51 focus formation relative to the parental line. Cells were irradiated with 8 Gy, fixed four hours later and stained as previously described[1]. Mean and s.d. of four replicates is shown.

Supplementary Figure 4. PARP1 loss in *BRCA1* mutant cells results in profound resistance to PARP inhibitors compared to other known resistance mechanisms.

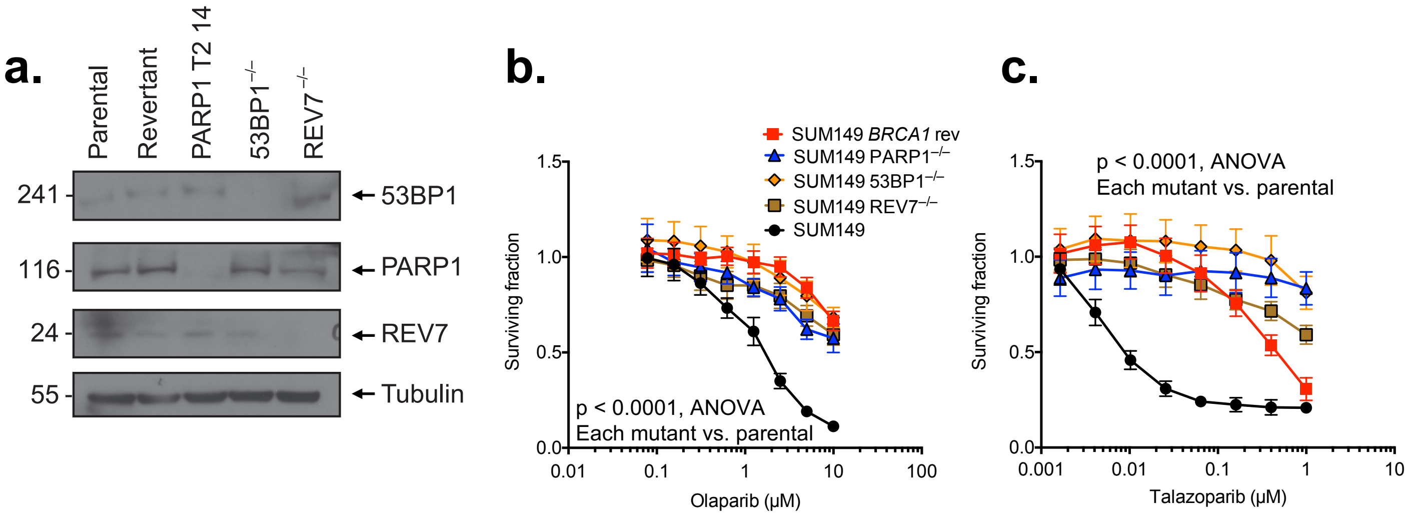

Supplementary Figure 4. PARP1 loss in *BRCA1* mutant cells results in profound resistance to PARP inhibitors compared to other known resistance mechanisms. (a) Western blot showing expression of 53BP1, PARP1 and REV7 protein in a series of isogenic series of SUM149 cell lines generated via CRISPR mutagenesis. Lysates from SUM149 cells (Parental) and clones generated by CRISPR-mediated reversion of the *BRCA1* mutation ("Revertant"), knockout of *PARP1*, *TP53BP1* (53BP1 clones) or *REV7* were blotted and analysed with the indicated antibodies. **(b)** Olaparib and **(c)** talazoparib survival assays for cell lines analysed in (a). Cells were plated in 384-well plates, exposed to drug for seven days and survival assayed using CellTiter Glo. All cell lines are significantly resistant to PARPi compared to parental SUM149 cells ($p < 0.0001$, ANOVA). Mean of 16 replicates plotted, error bars show s.d..

Supplementary Figure 5. PARP1 loss results in profound resistance to PARP inhibitors compared to other breast cancer cell lines.

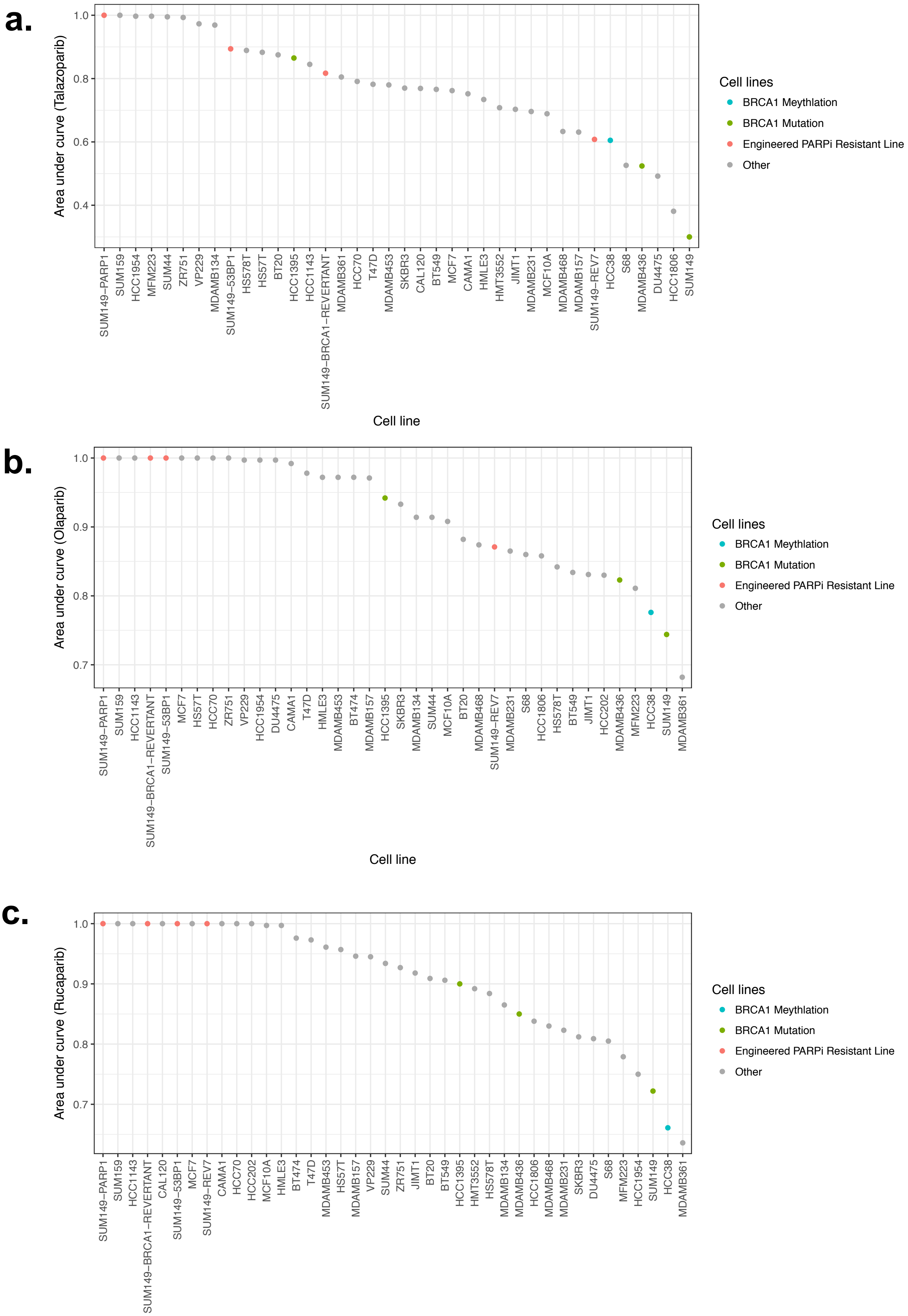

Supplementary Figure 5. PARP1 loss results in profound resistance to PARP inhibitors compared to other breast cancer cell lines. Waterfall plots showing **(a)** talazoparib, **(b)** olaparib or **(c)** rucaparib area under curve for a panel of breast cancer cell lines exposed to varying concentrations of talazoparib (0.5 – 1000 nM, eight concentrations).

Supplementary Figure 6 – Sensitivity to cisplatin and topoisomerase I inhibitors in *BRCA1;PARP1* mutant cells

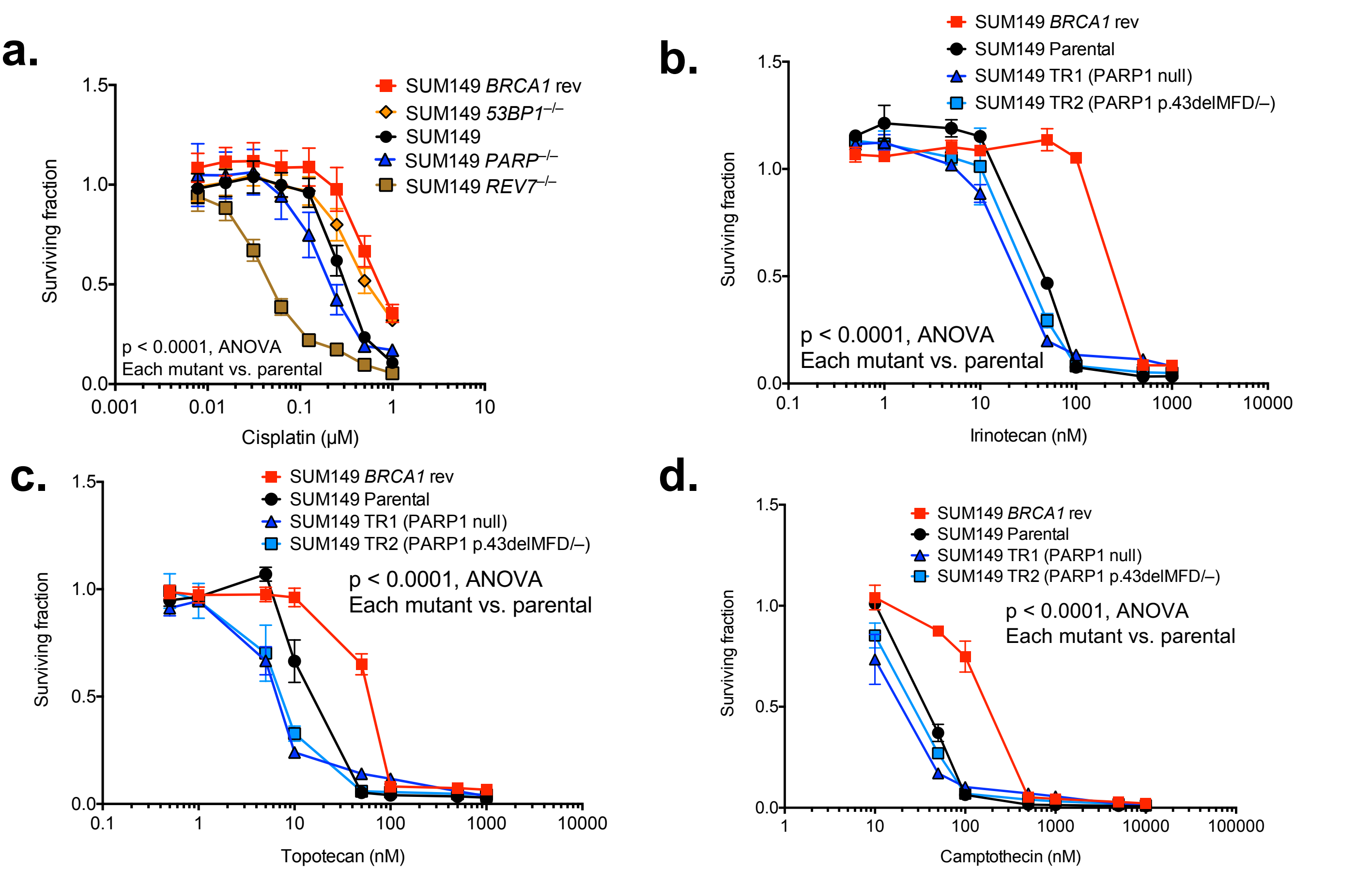

Supplementary Figure 6. Sensitivity to cisplatin and topoisomerase I inhibitors in *BRCA1;PARP1* mutant cells. **(a)** Differential sensitivity to cisplatin among PARPi resistance mechanisms. *BRCA1* reversion or 53BP1 loss causes resistance to cisplatin relative to the parental cells. However, the *PARP1* mutant clone has similar or slightly increased sensitivity relative to the parental cells and the *REV7* mutant has greatly increased sensitivity ($p < 0.0001$, ANOVA, all mutant-parental pairwise comparisons). Mean of 16 replicates plotted, error bars show s.d.. **(b)** *PARP1* mutant SUM149 cells TR1 and TR2 (blue) retain sensitivity to the topoisomerase I inhibitor irinotecan similar to the parental cells, unlike cells that have a reversion mutation in *BRCA1* (B1S*, red). Cells were exposed to the indicated concentrations of irinotecan for five days and survival assayed using CellTiter Glo. The mean and standard deviation of five replicates is plotted. Mean of five replicates plotted, error bars show s.d.. Similar results are seen for the topoisomerase I inhibitors topotecan **(c)** and camptothecin **(d)**.

Supplementary Figure 7 – PARP1 mutations in COV362 and MDA-MB-436 cells

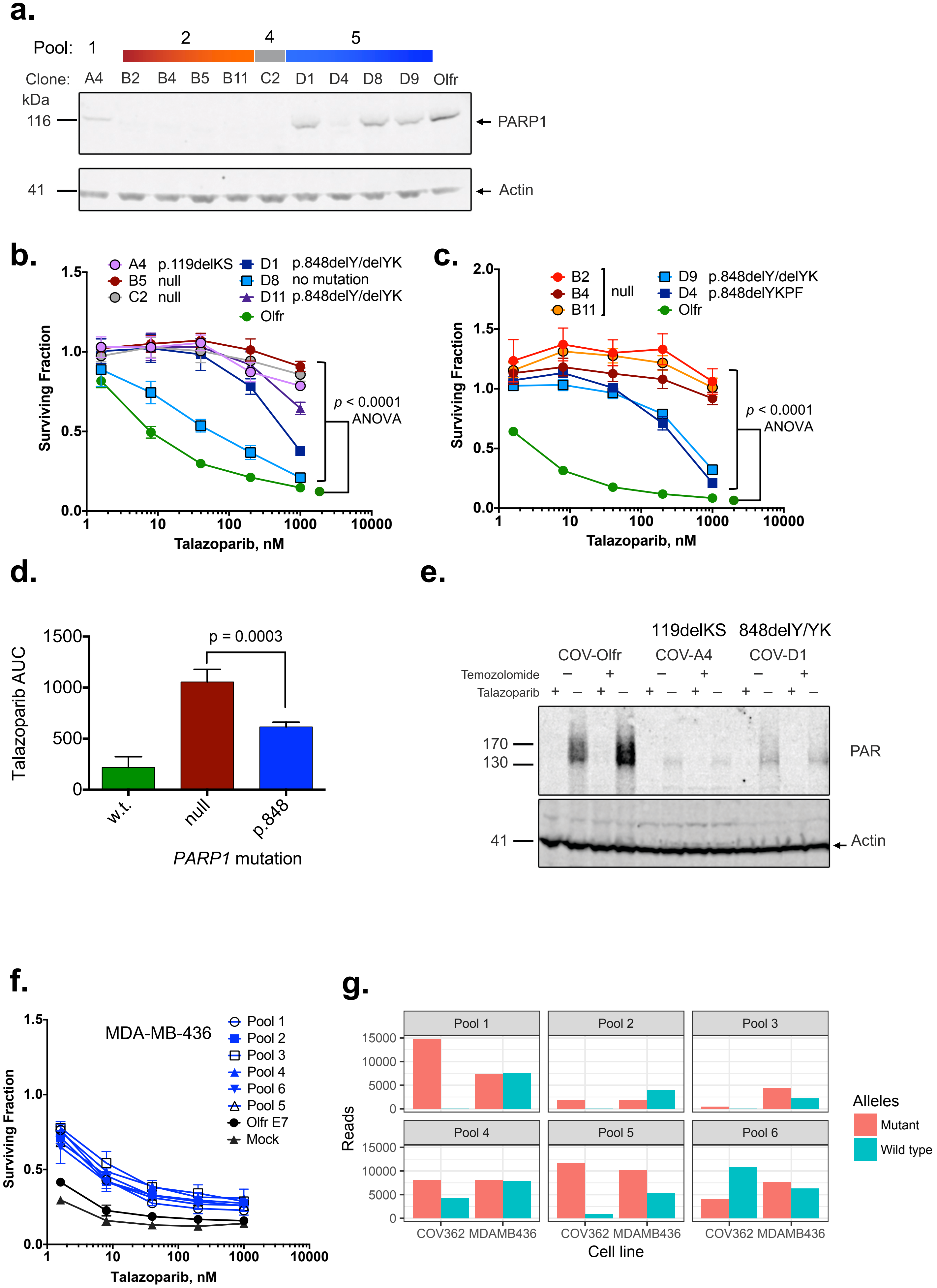

Legend overleaf

Supplementary Figure 7. PARP1 mutations in COV362 and MDA-MB-436 cells. (a) Western blot of PARP1 expression in cell lysates from clonal talazoparib-resistant COV362 cell lines (isolated from resistant cell populations shown in D). Olfr, COV362-Cas9 cells transduced with a control guide RNA targeting a mouse olfactory receptor (sequence not present in human genome). **(b, c)** Talazoparib survival curves for subcloned *PARP1* mutant COV362 lines. ANOVA $p < 0.0001$ for all mutants compared to Olfr control. Note the mild resistance in clone D8, in which a *PARP1* mutation was not identified. Mean of five replicates plotted, error bars show s.d.. Surviving fractions were calculated relative to DMSO exposed cells for each mutant. **(d)** Area under talazoparib survival curve calculated from data plotted in F and G, grouped by type of *PARP1* mutation. The extent of resistance in Y848 mutants is significantly lower than null mutants ($p = 0.0003$, t-test), but still significant compared to wild type cells. **(e)** Retention of limited cellular PARP activity in COV362 cells shown by western blot of lysates from cells treated with temozolomide and/or talazoparib as shown, probed with anti-PAR antibody. **(f)** Short term talazoparib resistance in MDA-MB-436 cells induced by *PARP1* sgRNA. MDA-MB-436-Cas9 cells were transduced with the indicated pools of PARP1 lentivirus, selected in puromycin and exposed to talazoparib for five days as shown. Cell viability was assessed using CellTiter Glo. Although resistance was observed in this assay ($p < 0.0001$, ANOVA, compared to mock or control sgRNA OlfrE7 for all pools), no long term resistant cells could be isolated. Mean of five replicates plotted, error bars show s.d.. **(g)** Ion Torrent sequencing of PARP1 target sites in COV362-Cas9 or MDA-MB-436-Cas9 cells transduced with PARP1 sgRNA pools and exposed to talazoparib as shown in (j). The number of reads deriving from mutant (red) or wild type (blue) alleles is shown for each pool and cell line.

Supplementary Figure 8. Mutation clusters and sequence coverage of dense PARP1 screen.

a.

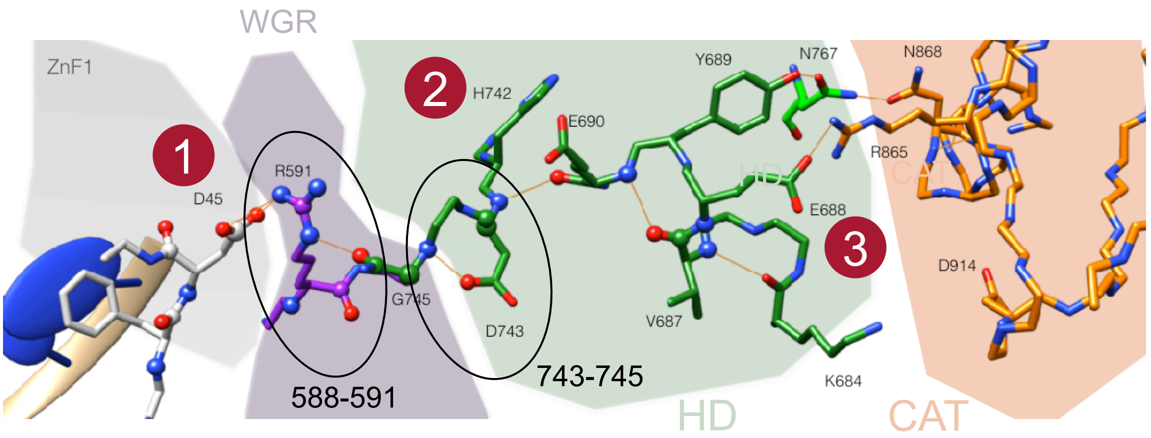

b.

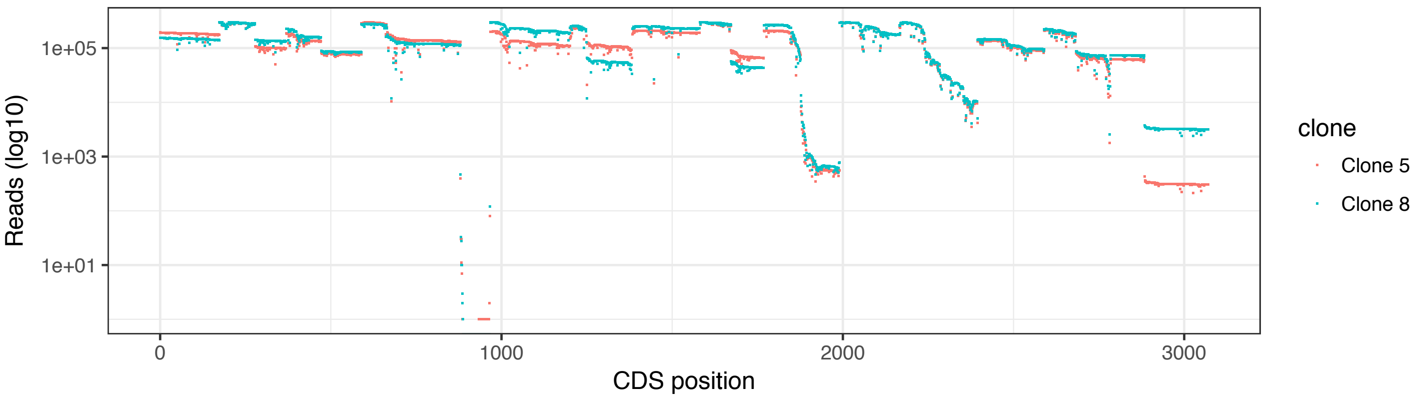

Supplementary Figure 8. Clusters and sequence coverage of dense PARP1 screen. (a) Positions of the clusters of mutations (black ellipses) outside the zinc finger domains identified in the dense tiling screen shown in relation to the proposed hydrogen bond network proposed to effect intramolecular communication within PARP1 (Figure 2g). **(b)** Per-base coverage shown for amplicon sequencing of *PARP1-GFP* RT-PCR products from clone 5 (red) and clone 8 (blue) in the dense tiling screen shown in Figure 4a, mapped onto the *PARP1* coding DNA sequence (CDS).

Supplementary Figure 9. Uncropped western blots

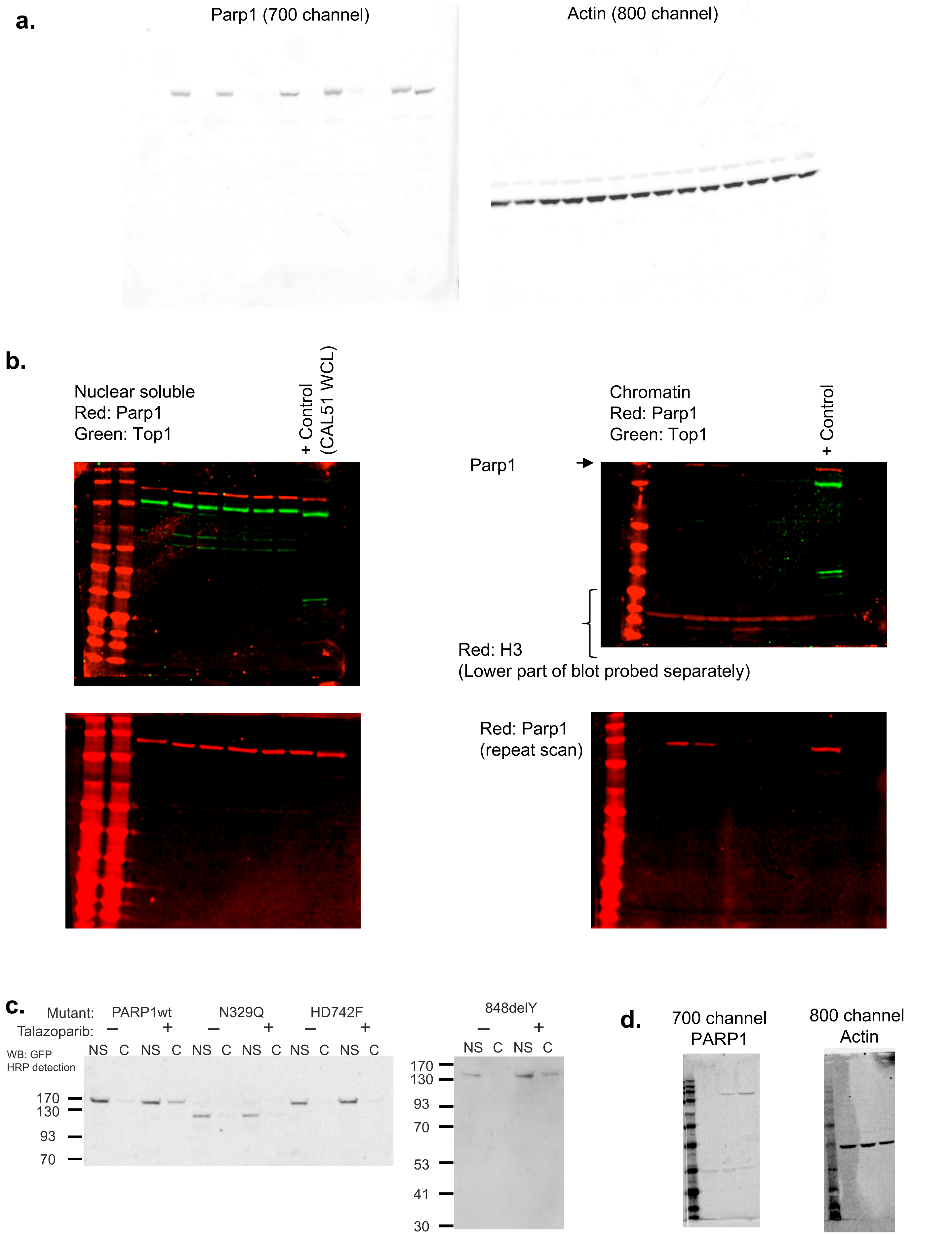

Supplementary Figure 9. Uncropped western blot images. (a) Figure 1c, (b) Figure 1e, (c) Figure 2e (d) Figure 3b.
