# Supplementary material for: Genome-wide and high-density CRISPR-Cas9 screens identify point mutations in PARP1 causing PARP inhibitor resistance
Source: Nat Commun. 2018 May 10;9:1849. doi: 10.1038/s41467-018-03917-2 (PMC5945626; doi:10.1038/s41467-018-03917-2)
Supplement: Supplementary file 2 — Description of Additional Supplementary Files [file 41467_2018_3917_MOESM2_ESM.pdf]

Description of Additional Supplementary Files

Supplementary Data 1. CRISPR sgRNA sequences used for the *PARP1* focused mutagenesis screen.

Supplementary Data 2. Translated protein alignments for the *PARP1* mutants isolated in the HeLa screen.

Supplementary Data 3. Ion Torrent sequencing mutation calls for the *PARP1* mutants isolated in the HeLa screen.

Supplementary Data 4. *PARP1* mutations in talazoparib-resistant SUM149 clones TR1 and TR2.

Supplementary Data 5. Guide sequences used in the *PARP1* dense library.

Supplementary Data 6. Primer sequences with Ion Torrent adapters for genotyping mutations in the dense *PARP1* mutagenesis screen.

Supplementary Data 7. *PARP1* mutations identified in talazoparib-resistant SUM149 *PARP1-tagGFP2* cells in the dense mutagenesis screen.

Supplementary Data 8. Primer and sgRNA target sequences used in this study.

Supplementary Data 9. Antibodies and working dilutions used in this study.
